# From the TRICE-2 Investigations to the TRACERS Mission

**DOI:** 10.1007/s11214-025-01178-2

**Published:** 2025-06-11

**Authors:** K. J. Trattner, J. LaBelle, O. Santolik, C. A. Kletzing, D. M. Miles, S. A. Fuselier, J. W. Bonnell, S. R. Bounds, I. Kolmasova, S. M. Petrinec, R. P. Sawyer, S. K. Vines, C. Moser-Gauthier, I. H. Cairns, T. K. Yeoman

**Affiliations:** 1 LASP, University of Colorado, Boulder, CO USA; 2 Department of Physics and Astronomy, Dartmouth College, Hanover, NH USA; 3 Department of Physics and Astronomy, University of Iowa, Iowa City, IA USA; 4 Southwest Research Institute, San Antonio, TX USA; 5 University of Texas at San Antonio, San Antonio, TX USA; 6 Space Sciences Laboratory, University of California, Berkeley, CA USA; 7 Lockheed Martin Advanced Technology Center, Palo Alto, CA USA; 8 School of Physics, University of Sydney, Sydney, New South Wales 2006 Australia; 9 Department of Physics and Astronomy, University of Leicester, Leicester, UK; 10 University of New Hampshire, Durham, NH USA; 11 Department of Space Physics, Institute of Atmospheric Physics of the Czech Academy of Sciences, Prague, Czechia; 12 Faculty of Mathematics and Physics, Charles University, Prague, Czechia

**Keywords:** Magnetic reconnection, Reconnection location, Plasma entry into magnetosphere, Precipitating ions, Boundary layer

## Abstract

On the morning of December 8, 2018, two sounding rockets were launched into the northern hemisphere cusp region to investigate the spatial and temporal nature of cusp structures. The two rockets, designated Twin Rockets to Investigate Cusp Electrodynamics 2 (TRICE-2), consisted of a high- and a low-flyer rocket launched two minutes apart. The TRICE-2 mission was a pathfinder for the upcoming Tandem Reconnection and Cusp Electrodynamics Reconnaissance Satellites (TRACERS) mission and carried almost identical payloads to those proposed for the twin spacecraft of the TRACERS mission. Results from the TRICE-2 mission are summarized, including observed cusp features (low energy ions in the cusp, overlapping cusp ion dispersions and cusp ion signatures) and the connection of the cusp structures to ionospheric convection cells, provided by SuperDARN radar observations, to show the advantages of coordinated space and ground-based observations. A description is provided for how these results – and those of other experiments which made measurements of particles and waves in the cusp and in the dayside magnetosphere – have guided the science objectives of the TRACERS mission.

## Introduction

After decades of research, it has been established that magnetic reconnection (e.g., Dungey 1961, 1963; Gonzalez and Mozer 1974; Paschmann et al. 1979; Sonnerup et al. 1981; Cowley and Owen 1989) is the dominant process active at the Earth’s magnetopause that is responsible for the transfer of mass, energy and momentum from the thermal solar wind plasma (heliosphere) into the terrestrial space environment. Magnetic reconnection also plays a decisive role in defining what plasma populations are present within the magnetospheric cusps (Fuselier and Lewis 2011; Cassak and Fuselier 2016; Smith and Lockwood 1996; Gosling et al. 1990). At the Earth’s magnetopause, this reconnection process where the interplanetary magnetic field (IMF) merges with the geomagnetic field has been documented for all orientations of the IMF (e.g., Sonnerup 1974; Crooker 1979; Luhmann et al. 1984; Gonzalez and Mozer 1974; Cowley and Owen 1989; Gosling et al. 1991; Trattner et al., 2006; Fuselier and Lewis 2011).

Dedicated missions to study magnetic reconnection, e.g., the NASA/Magnetospheric Multiscale Mission (MMS) (Burch et al. 2016), observe reconnection in situ at the magnetopause and have revealed many details of the process (e.g., Burch et al. 2016; Cassak and Fuselier 2016; Fuselier et al. 2017b; Webster et al. 2018). However, the MMS mission is only able to observe magnetic reconnection directly for short periods of time when the satellites encounter the very small target area (the X-line) at the magnetopause. That leaves many aspects of magnetic reconnection unresolved, e.g., the global spatial or temporal nature of the process (either spatially separated X-lines at the magnetopause or X-lines that briefly cease to exist), the variation of the reconnection rate associated with temporal reconnection, and various dynamic features observed in the cusp and in the dayside magnetosphere in general.

In addition to in situ observations of magnetic reconnection at the magnetopause, the cusps are another prominent magnetospheric region in which to study aspects of magnetic reconnection (e.g., Burch 1972; Newell and Meng 1989, 1992; Woch and Lundin 1992; Lavraud et al. 2005; Trattner et al. 2002; Merka et al. 2005). In the cusps all geomagnetic field lines that define the magnetopause converge into two relatively narrow funnels, one at high northern latitudes and one at high southern latitudes. These funnels allow solar wind ions to have direct access to the low altitude ionosphere. Therefore, all processes that occur on the magnetopause surface also leave signatures in the precipitating ion distributions observed in the cusps (e.g., Cowley et al. 1991; Cowley and Lockwood 1992; Lockwood and Smith 1994; Oieroset et al. 1997; Fuselier et al. 1999; Trattner et al. 2005a; Escoubet et al. 2008). Depending on their specific altitudes, polar orbiting satellites (e.g., DMSP, Polar, Cluster) can remain in the cusp for hours and continuously observe precipitating magnetosheath ions and their respective reconnection signatures to address these more global aspects of magnetic reconnection.

Magnetic reconnection is also studied using sub-orbital sounding rockets that are launched into the cusp (e.g., Frederick-Frost et al. 2007; Di Mare et al. 2021; Sawyer et al. 2021; Fuselier et al. 2022). In preparation for the upcoming Tandem Reconnection and Cusp Electrodynamics Reconnaissance Satellites (TRACERS) mission, two sounding rockets were launched to study the spatial and temporal nature of magnetic reconnection. The sounding rockets, designated Twin Rockets to Investigate Cusp Electrodynamics 2 (TRICE-2) were part of NASA’s Grand Challenge Initiative and were launched from the Andoya Space Flight Center in Norway on December 08, 2018. Due to their close ties to the TRACERS mission, the two rockets carried nearly identical payloads to the anticipated instruments on TRACERS and were launched with a temporal separation of two minutes into the Earth’s northern magnetospheric cusp. The High-Flyer rocket, designated HF52003, reached an altitude of 1042 km and was launched at 08:26 UT, while the Low-Flyer rocket, designated LF52004, launched at 08:28 UT and crossed the cusp at an altitude of 756 km shortly after its high-flying counterpart.

It is the purpose of this review to summarize the results of the TRICE-2 “pathfinder” mission, assimilate the results with the findings of earlier cusp missions, and summarize and discuss the available observations and methodologies to be used for the upcoming TRACERS mission. Additionally, the review outlines avenues of auxiliary or complementary science objectives that TRACERS can directly address and synergies with other available ground-based and satellite measurements.

## Cusp Ion Dispersions – Spatial or Temporal

Satellites that cross the magnetospheric cusp regions at high altitudes encounter newly reconnected geomagnetic field lines with precipitating magnetosheath ions arriving from the magnetopause reconnection location (the X-line), and upward propagating mirrored magnetosheath ions that return to the observing satellite from the ionospheric mirror points. Precipitating ions in the cusps feature a distinctive velocity dispersion profile caused by the different flight times of the ions from where they originally crossed the magnetopause along with the simultaneous convection of the open geomagnetic field line with the solar wind. For a satellite crossing the cusp under southward IMF conditions, the cusp ion dispersion resembles a single saw-tooth with the highest velocity ions arriving at the lowest magnetic latitudes while slower magnetosheath ions, that need more time to arrive at the satellite, are convected to higher latitudes. This characteristic decreasing ion dispersion feature was predicted by Rosenbauer et al. (1975) and later observed by Shelley et al. (1976).

The above-described precipitation and convection model causes a single decreasing ion-energy dispersion event, which also represents the signature of a dayside magnetic reconnection process that is continuous and steady-state. Such single ion dispersion events are rare in cusp observations and only a few have been found (e.g., Escoubet et al. 2008). Their scarcity indicates that steady-state magnetic reconnection is not a dominant feature at the Earth’s magnetopause. However, while they do exist, it is currently unknown what outside solar wind/IMF conditions or internal conditions, e.g., the location of magnetic reconnection at the magnetopause, could cause such a steady-state behavior for the magnetic reconnection process.

While magnetic reconnection at the Earth’s magnetopause is rarely a continuous process, it is highly variable and seem to switch on and off periodically (pulsing), even when active at the same location at the magnetopause. The reason for this pulsed behavior of magnetic reconnection is unknown but dramatically influences the cusp ion profile observed by a satellite. As described in the pulsed reconnection model (e.g., Cowley and Owen 1989; Lockwood and Smith 1989, 1990; Lockwood et al. 1998), when magnetic reconnection at the magnetopause stops for several minutes (e.g., Trattner et al. 2015), it significantly slows down convection of open magnetic field lines through the cusp. When magnetic reconnection restarts, the newly opened geomagnetic field line convecting across the cusp is located next to a geomagnetic field line that opened minutes ago. Magnetosheath ions injected onto the open geomagnetic field lines precipitate into the cusp. For the magnetic field line that opened minutes ago, the time-of-flight effect from the reconnection site leads to lower velocity ions arriving at the cusp satellite. In contrast, for the newly opened magnetic field line, only the fastest recently injected ions can reach the satellite. A cusp satellite crossing this boundary between the newly opened field lines and field lines opened long ago will observe a sharp step in the ion energy dispersion profile. This step (e.g., Newell and Meng 1991; Escoubet et al. 1992) in the ion-energy profile is either a “Step-Up” in energy, if the field line convection speed is overtaking the satellite and providing access to newly opened magnetic field lines, or a “Step-Down” in energy, if the satellite overtakes the field line convection and reaches open cusp magnetic field lines that have been open longer.

Therefore, what ion distributions are observed in the cusp at a specific location by a satellite depends mainly on how long the current magnetic field line is open (e.g., Trattner et al. 2015), on the reconnection location at the magnetopause (distance to the plasma injection point) (e.g., Trattner et al. 2007, 2021, Petrinec et al. 2014, Fuselier et al. 2018, 2022) and the velocity distribution of the plasma injected onto the newly opened magnetic field line.

**Fig. 1:**
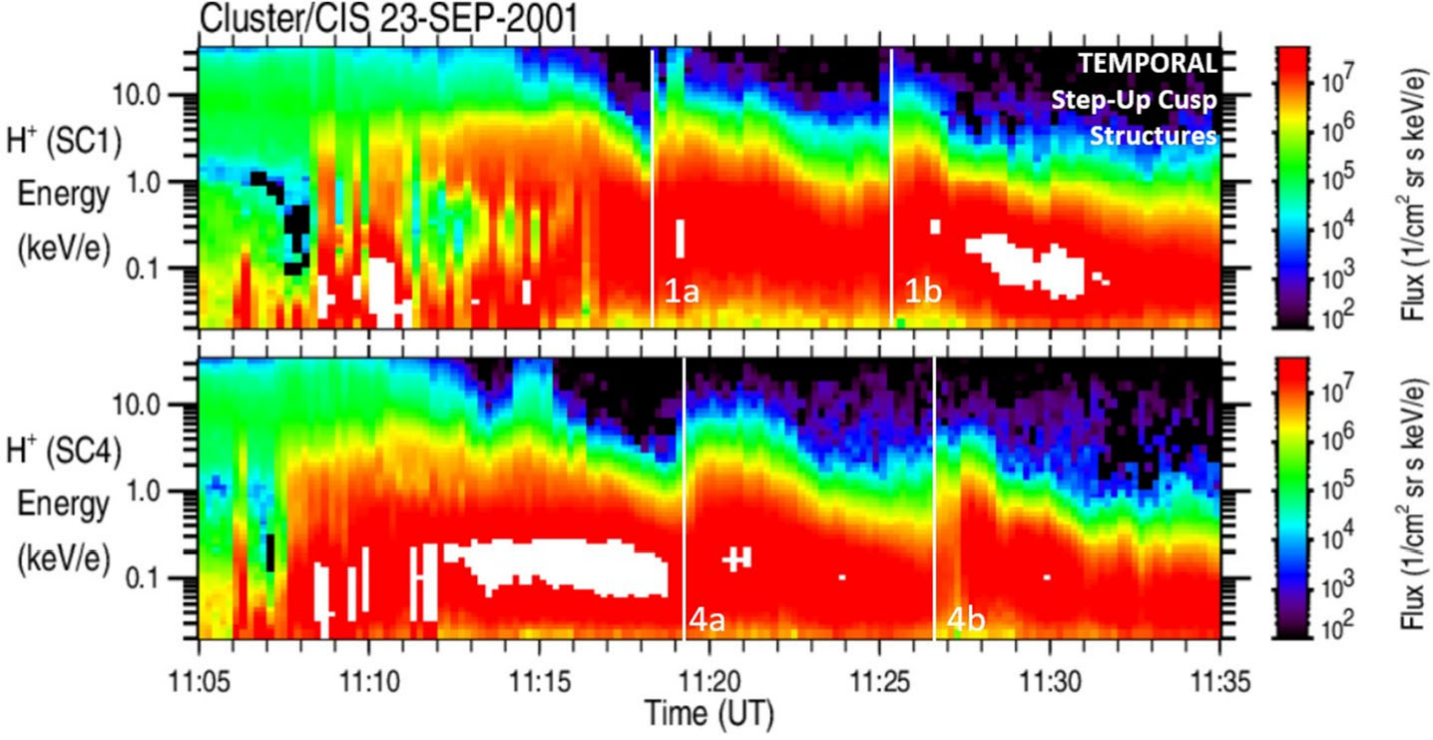
H^+^ omnidirectional ion flux observations, observed by the Cluster/CIS instrument during the cusp crossing on September 23, 2001, for the SC1 (top panel) and SC4 (bottom panel) satellites. The cusp ion-energy dispersion profile shows several “Temporal” Step-Up cusp structures (marked by vertical white lines) associated with pulsed reconnection at the magnetopause. Adapted from Trattner et al. (2008)

Figure 1 shows the Cluster/CIS northern cusp crossing on September 23, 2001. Plotted are H^+^ omnidirectional ion-energy flux measurements for spacecraft SC1 (top panel) and SC4 (bottom panel). The Cluster satellites crossed the cusp in about 40 minutes at an altitude of 4-5 Earth Radii (R_E_). With SC4 being the leading satellite, both satellites proceeded poleward through the cusp along roughly the same trajectory and only minutes apart; a configuration that is very similar to the mission design set for the TRACERS mission. The Cluster SC1 and SC4 satellites crossed into the cusp and encountered precipitating magnetosheath ions at about 11:12 UT and 11:10 UT, respectively. Both satellites observed typical cusp ion-energy dispersion profiles with two Step-Up cusp structures marked by vertical white lines (a, b). The trailing satellite SC1 encountered the Step-Up cusp structures at about 11:18.15 UT (1a) and 11:25.10 UT (1b) while the leading satellite SC4 located at higher latitudes encountered the Step-Up cusp structures at about 11:19.10 UT (4a) and 11:26.40 UT (4b). A series of Step-Up cusp structures encountered by multiple satellites sequentially at different latitudes is the classical signature known as temporal cusp structures caused by pulsed reconnection at the magnetopause (e.g., Cowley et al. 1991; Lockwood and Smith 1989, 1990; Smith et al. 1992; Farrugia et al. 1998; McWilliams et al. 2004; Escoubet et al. 2006; Trattner et al. 2008).

**Fig. 2:**
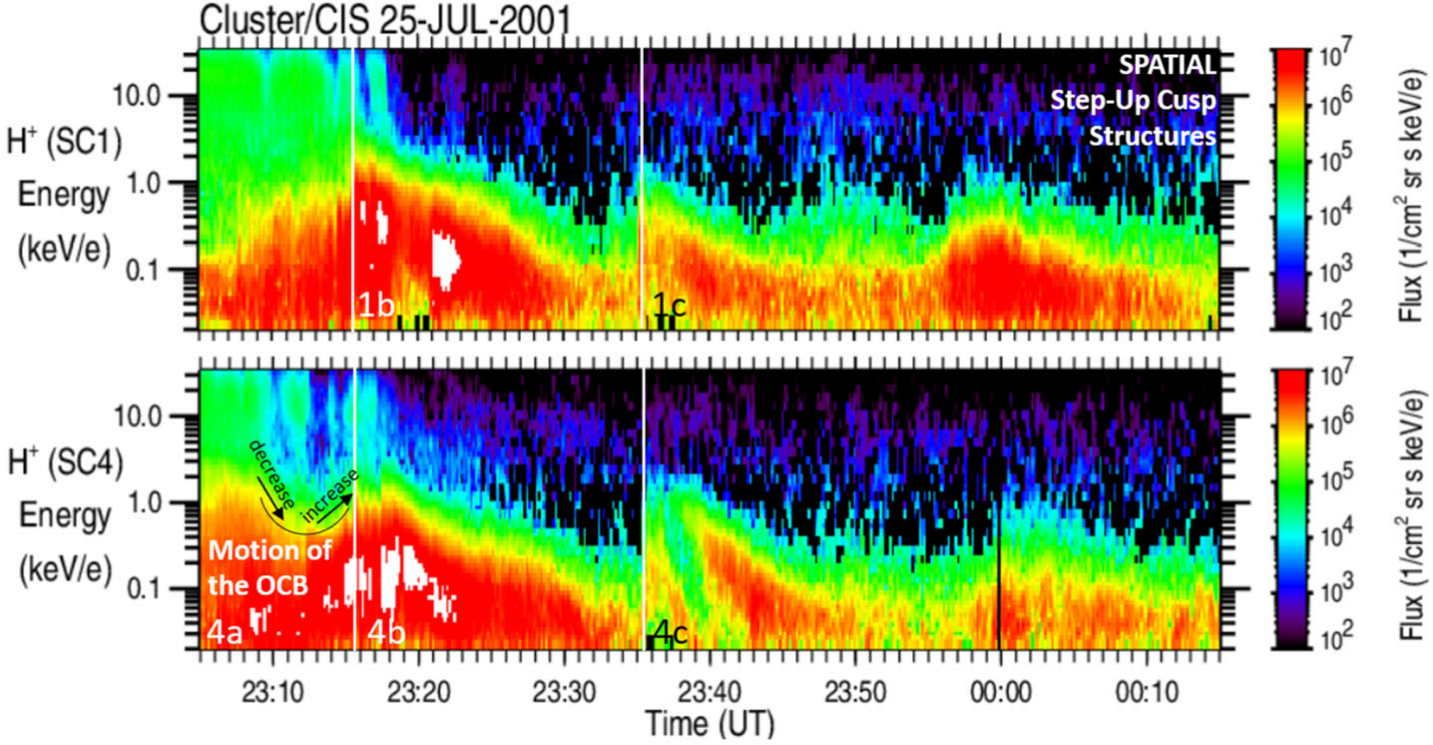
H^+^ omnidirectional ion flux observations, observed by the Cluster/CIS cusp crossing on July 25, 2001, for the SC1 and SC4 spacecrafts. The cusp ion dispersion profile shows the consequence of a motion of the open-closed field line boundary (OCB) and several “Spatial” Step-Up cusp structures (marked by white vertical lines) associated with spatially separated flux tubes with their own time history since reconnection occurred at the magnetopause. Adapted from Trattner et al. (2005a)

Figure 2 shows the Cluster/CIS northern cusp crossing on July 25, 2001. The layout of Fig. 2 is the same as in Fig. 1. As in the previous case, SC4 is the leading satellite; it crossed into the cusp at 23:09 UT where it first encountered the classical decreasing cusp ion-energy profile (4a) before the energy of the precipitating ions slowly increases, again. This continuous change in the cusp ion-energy profile (not Step like) was analyzed with the help of ionospheric convection cells provided by the SuperDARN radar arrays and interpreted as a motion of the Open-Closed-Field Line Boundary (OCB) with respect to the magnetic footpoints of the observing satellite in the cusp (Trattner et al. 2003). The entire ionospheric convection cell connected to the cusp field lines reconfigured, which moved the OCB and shortened the convection path of the open magnetic field line from the OCB to the location of the Cluster satellite in the cusp. Cluster therefore encountered open magnetic field lines that reconnected more recently. Located on these recently opened magnetic field lines, only ions with higher energy can reach the satellite which is expressed in the gentle turnaround of the ion-energy dispersion profile.

At about 23:15.40 UT, the precipitating cusp ions on SC4 reached their maximum energy, marked by a vertical white line (4b), followed by the classical decreasing velocity dispersion. This is also the time when SC1 crossed into the cusp and observed the classical decreasing velocity dispersion (1b). Both satellites subsequently observed several Step-Up cusp structures with only the first set marked by white vertical lines (1c and 4c).

These two events highlight the difficulties in the interpretation of cusp structures and how to distinguish their spatial or temporal nature. Overall, the ion-energy dispersions in Fig. 1 and 2 are very similar. Both cusp crossings show a series of Step-Up cusp structures. However, while it has been shown that the cusp structures in Fig. 1 are of a temporal nature and convect with the solar wind flow to higher magnetic latitudes, it has been determined that the cusp structures in Fig. 2 are of a spatial nature (e.g., Onsager et al. 1995; Wing et al., 2001; Trattner et al. 2002, 2003, 2005a). Under normal circumstances, these spatial structures would appear at the same magnetic latitude. However, for this event, the specific observational conditions caused a complication. The Cluster satellites cross the cusps at high altitudes with characteristic crossing durations of about 1 hour, while at the same time magnetic reconnection is a very dynamic process causing changes in the magnetic topology and the respective ionospheric convection pattern on a time scale of minutes. This caused a puzzling detail in Fig. 2, where the Step-Up cusp structures 1c and 4c seem to be observed at the same time on both satellites while SC1 and SC4 are located at different magnetic latitudes inside the cusp.

**Fig. 3:**
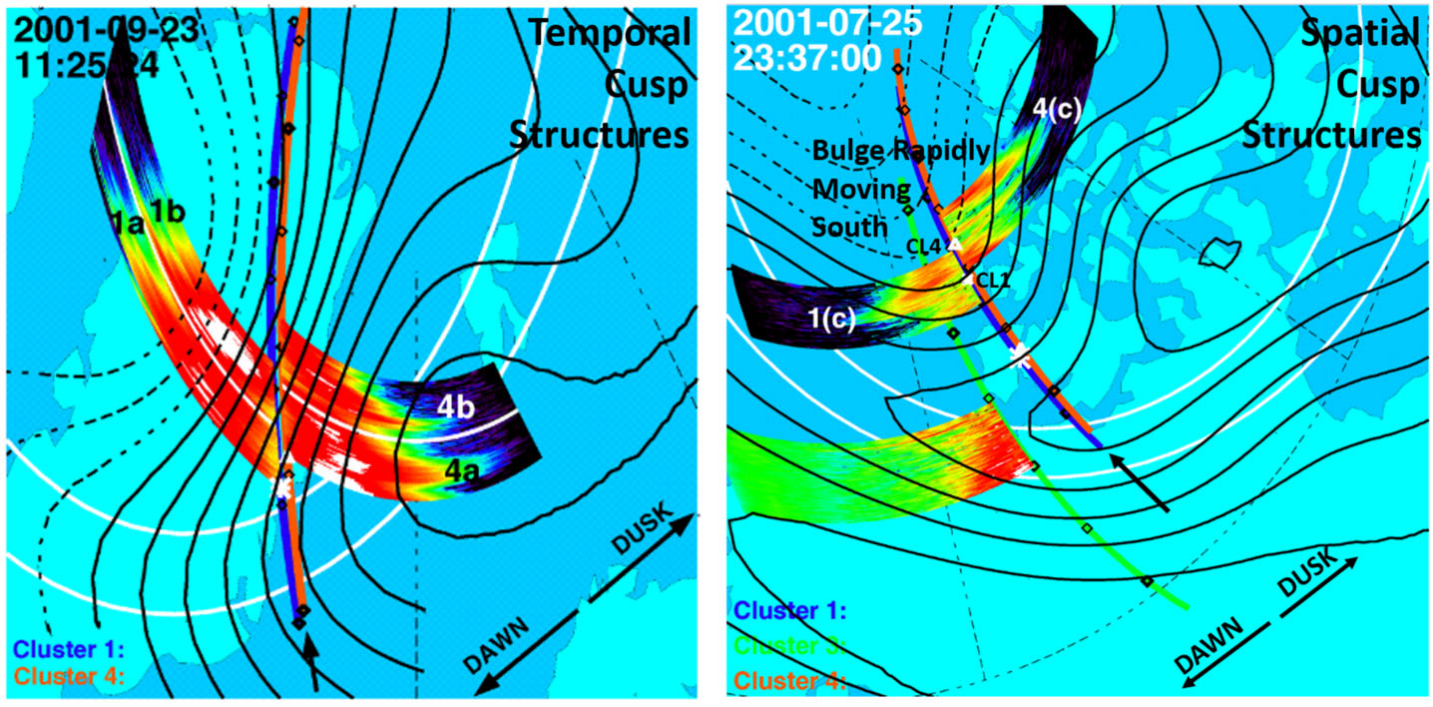
The Dawn (black dashed lines) and Dusk (black solid lines) ionospheric convection cells in the northern polar region, determined from observations of the SuperDARN radar array. The left panel shows a convection cell configuration for temporal cusp structures observed on 23 September 2001, while the right panel shows the configuration for spatial cusp structures observed on 25 July 2001. Overlayed are the magnetic foot points (blue and red curves) of the Cluster SC1 and SC4 spacecraft trajectories, respectively. Along the magnetic foot points, sections of the SC1 and SC4 ion color spectrogram in the cusp are shown, centered around the location of each spacecraft at the time of observation. The white lines show an average location of the auroral oval

The event in Fig. 2 was determined to be a spatial cusp structure by including into the analysis contextual observations provided by the SuperDARN radar arrays. These observations are expressed as ionospheric convection cells. Figure 3 shows the ionospheric convection cells for the two events discussed in Fig. 1 and 2. Black solid lines and black dashed lines represent the dusk and dawn ionospheric convection cells, respectively, plotted around the magnetic foot points of the Cluster satellites. These magnetic foot points are marked as blue and red lines for SC1 and SC4, respectively. Along the lines representing the magnetic foot points, several minutes of data from the color spectrogram shown in Figs. 1 and 2 are plotted, centered around the location of the satellites at that time at their respective magnetic foot point trajectories. The satellite’s location is marked with white solid lines in the color spectrograms.

The left panel in Fig. 3 shows the ionospheric convection cells and the location of the SC1 and SC4 satellites in the cusp at 11:25.24 UT on September 23, 2001. Both satellites are located in the dusk convection cells and move poleward along the poleward directed ionospheric convection flow. At the event time, SC1 (along the blue trajectory) has encountered cusp structure 1b which is marked by a white solid line in the color spectrogram. At the same time, SC4 (the red trajectory) is poleward of SC1 and in between cusp structures 4a and 4b. The color spectrogram for SC4 along the red trajectory shows that SC4 will encounter cusp structure 4b in about a minute and at an invariant magnetic latitude about 1 degree higher compared to the location of SC1. The ionospheric convection velocity provided by the SuperDARN radar data of about 1°/min matches the observation of the convecting cusp structures observed at SC1 and SC4 (Trattner et al. 2003), and confirms the temporal nature of these structures, caused by pulsed reconnection at the magnetopause.

The right panel in Fig. 3 shows the ionospheric convection cells and the location of the SC1 and SC4 satellites in the cusp at 23:37.00 UT on July 25, 2001. In contrast to the event shown on the left side of Fig. 3, the trajectories of SC1 and SC4 are not moving along the ionospheric convection direction, but across it. In addition, a time series of the ionospheric convection cell’s locations during the Cluster cusp crossings (not shown) indicated a sudden and major change in the location of the boundary separating the two convection cells. At the event time in the right panel in Fig. 3, a bulge has formed in the dawn convection cells that expanded rapidly southward and moved across the cusp locations of both Cluster satellites. Satellites SC1 and SC4 simultaneously switch from being magnetically connected to the dusk convection cell to being immersed in the spatially separated dawn convection cell and both satellites observe spatial Step-Up cusp structures at the same time, despite being located at different magnetic latitudes.

**Fig. 4:**
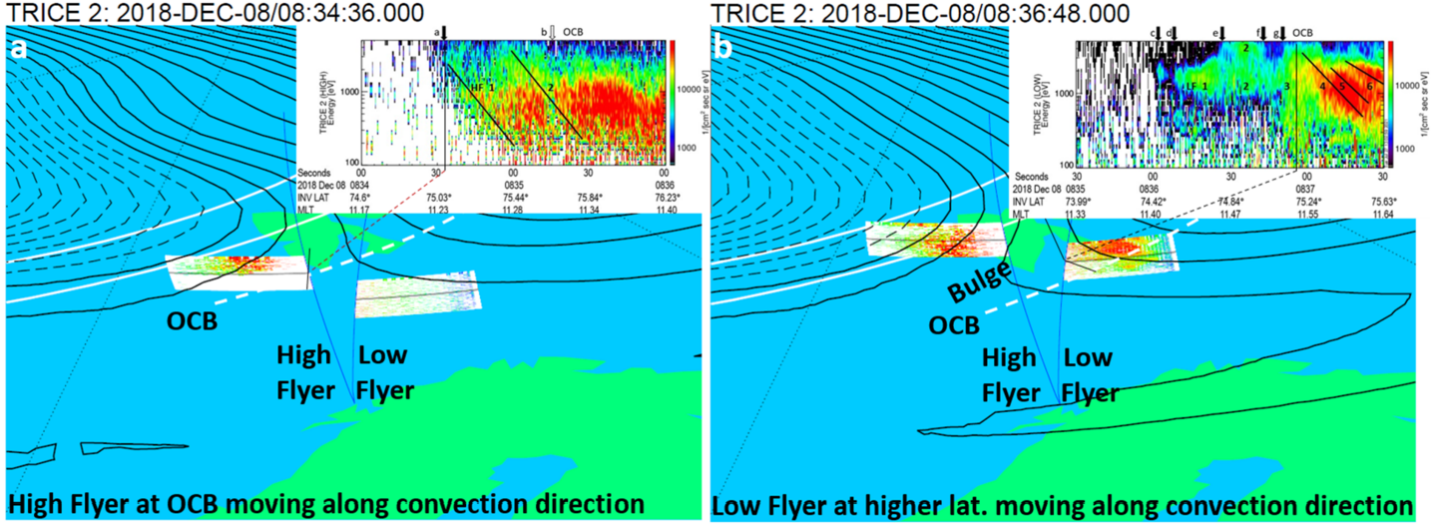
The Dawn (black dashed lines) and Dusk (black solid lines) ionospheric convection cells in the northern polar region, determined from observations of the SuperDARN radar array. The left panel shows the convection cell configuration for the time when the Trice-2 High Flyer entered the cusp while the left panel showed the configuration when the Trice-2 Low Flyer trajectory began to line up with the ionospheric convection direction. Overlayed are the magnetic foot points (light blue curves) of the trajectories of the TRICE-2 sounding rockets as they crossed the northern cusp region, the location of the open-closed field line boundary (OCB) (dashed white lines) and the average location of the auroral oval (solid white lines). The insets in the panels show the ion color spectrograms observed by the TRICE-2 sounding rockets in the northern cusp. Adapted from Trattner et al. (2024)

Including the ionospheric convection cell pattern for context has also proven beneficial for the interpretation of the TRICE-2 cusp crossings (e.g., Trattner et al. 2024). Figure 4 shows the TRICE-2 cusp crossings together with the ionospheric convection cells provided by SuperDARN for the time 08:34.36 UT (a: left panel) when the High-Flyer crossed into the cusp and at 08:36.48 UT (b: right panel) while the trajectory of the Low-Flyer located already in the cusp started to align with the ionospheric convection flow. The layout of Fig. 4 is the same as in Fig. 3 with the exception that the magnetic footpoints for the two rockets are drawn as thin blue lines and labeled accordingly.

In contrast to the above discussed Cluster example and the proposed trajectories for the TRACERS satellites, the TRICE-2 rockets did not proceed poleward along the same trajectory but drifted apart as they entered the cusp. Despite their temporal and spatial proximity, this small longitudinal separation causes significant differences in the observed ion-energy dispersions at the two rockets (see insets of the respective color spectrograms in the panels) which was further complicated by another bulge forming in the dusk convection cell and moving across the path of the two rockets.

The High-Flyer crossed into the center of the cusp, moving along the prevailing ionospheric convection direction in that location and observed the classical saw-tooth dispersion profile (rocket locations at that time marked by solid black lines in the color spectrogram insets). The Low-Flyer drifted to the east and crossed perpendicular to the ionospheric convection flow into the dusk convection cell. The Low-Flyer crossed the OCB several times, most likely caused by the motion of the bulge, before reaching a latitude where the ionospheric convection flow turned poleward and more aligned with the rocket’s trajectory. That transition is depicted in panel (b) in Fig. 4 and marked in the inset by a solid black line. From there on, the Low-Flyer also observed the classical decreasing dispersion profile for several overlapping ion-energy dispersions, marked by black lines (4, 5, 6) and identified by local maxima in the ion flux measurements, which indicates the presence of multiple X-lines at the magnetopause (e.g., Lee and Fu 1985; Fuselier et al. 2018, 2022; Trattner et al. 2012; Vines et al. 2017).

These examples show that convection in the cusp is not a simply steady poleward flow but shows surprising internal dynamics with changing flow patterns and motions of the OCB, all of them influencing the appearance, characteristics and interpretation of cusp structures observed by satellites and sounding rockets. The inclusion of context measurements like the ionospheric convection cells in the interpretation of the more complicated cusp structures proves to be very useful, especially when there are differences in multi-point observations during near-conjunction events.

While the Cluster mission consists of four high-altitude satellites that cross the cusp region slowly over about 1 hour and are overtaken by the open field line convection in the cusp, the TRACERS mission will cross the entire cusp in about 1-2 minutes and will be traversing the cusp in approximately the opposite direction of the convecting field line in the cusp for southward IMF conditions. In addition, the TRACERS spacecraft will follow each other along the same trajectory and will not have the added complication of two different trajectories like those of the TRICE-2 rockets. TRACERS will therefore provide true snapshots of the cusp ion-energy dispersion profiles as they are at that moment and will be less affected by changes in the convection flow pattern causing additional features. These circumstances will make the separation of spatial and temporal cusp structures easier by simply presenting the ion flux observations versus invariant magnetic latitude and documenting if cusp structures moved or appear at the same location. In addition, the high cadence of cusp observations by TRACERS will provide insights into additional features of magnetic reconnection which were previously inaccessible, e.g., single cusp ion dispersions caused by continuous steady-state magnetic reconnection at the magnetopause. This elusive feature, usually distorted by temporal effects, will appear very distinct in the snapshot cusp ion-energy profiles and will provide insights into the specific conditions required for steady-state reconnection to be active at the magnetopause. All these simplifications notwithstanding, the use of radar and other ground-based and satellite observations will be invaluable for determining the global context of the TRACERS cusp observations and helping to untangle changes in the external conditions during the short cusp crossing.

## Overlapping Cusp Dispersions

As mentioned above, the magnetic topology of the cusp regions causes any process occurring at the Earth’s magnetopause to leave a signature of that process in the cusp ion-energy dispersion profile. In addition to the common “Step-Up” and “Step-Down” dispersion profiles, sometimes overlapping ion dispersions are observed in the cusps (e.g., Lockwood 1995; Trattner et al. 1998, 2012, Fuselier et al. 1997, 2018; Pitout et al. 2012; da Silva et al. 2022, 2024). This specific cusp feature is generally interpreted as the signature for multiple X-lines present simultaneously at the Earth’s magnetopause (e.g., Lee and Fu 1985; Fuselier et al. 2011, 2018, 2022; Vines et al. 2017; Trattner et al. 2012, 2024). While some studies report a preference of the occurrence of multiple X-lines during southward IMF conditions together with a large IMF B_Y_ component (e.g., da Silva et al. 2024), their overall occurrence for different solar wind conditions is still unknown. However, the simultaneous occurrence of multiple X-lines at the magnetopause has a significant impact on the mass, energy, and momentum transport into and through the magnetosphere. The TRACERS mission with its high cadence of cusp observations will establish the relative occurrence rate of multiple X-lines at the magnetopause and determine the associated solar wind and IMF conditions.

**Fig. 5:**
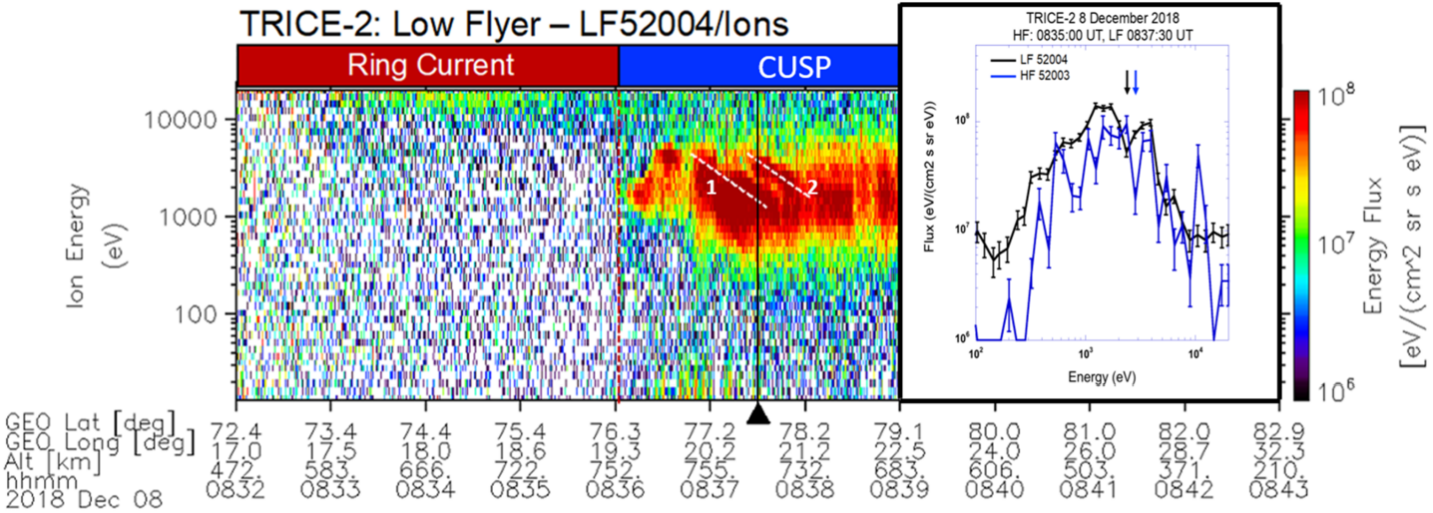
TRICE-2 (Low-Flyer) ion color spectrogram during the crossing of the northern cusp. The ion spectrometer on the rocket observed overlapping ion-energy dispersions (1 and 2), the signature of multiple X-lines presents at the magnetopause. Adapted from Fuselier et al. (2022)

Figure 5 shows the ion color spectrogram of the TRICE-2 Low-Flyer during the cusp crossing on December 8, 2018. As seen in Fig. 4, the Low-Flyer drifted off to the east and crossed into the dusk ionospheric convection cell which caused the highly structured cusp entry profile (e.g., Trattner et al. 2024). As the Low-Flyer progressed poleward and its trajectory aligned with the poleward convection flow, the ion instruments on both rockets observed the classical decreasing ion-energy dispersion. At that time, the Low-Flyer also observed overlapping ion-energy dispersions (white dashed lines marked as 1 and 2 in Fig. 5) indicating a secondary temporary X-line at the magnetopause (Fuselier et al. 2022; Trattner et al. 2024).

The inset at Fig. 5 shows the flux versus energy profile for the High-Flyer (blue line) and the Low-Flyer (black line) at the time marked in the color spectrogram by a black triangle when both rockets were at the same geomagnetic latitude. At that location, there are relative flux minima observed at 2.2 keV and 2.7 keV for the Low-Flyer and the High-Flyer, respectively. These local flux minima occur at the same geomagnetic latitude and about 2 minutes apart in time, in between the two overlapping ion energy dispersions. These observations were interpreted as quasi-stationary magnetic reconnection in time and location for the 2-minute observation time (Fuselier et al. 2022).

**Fig. 6:**
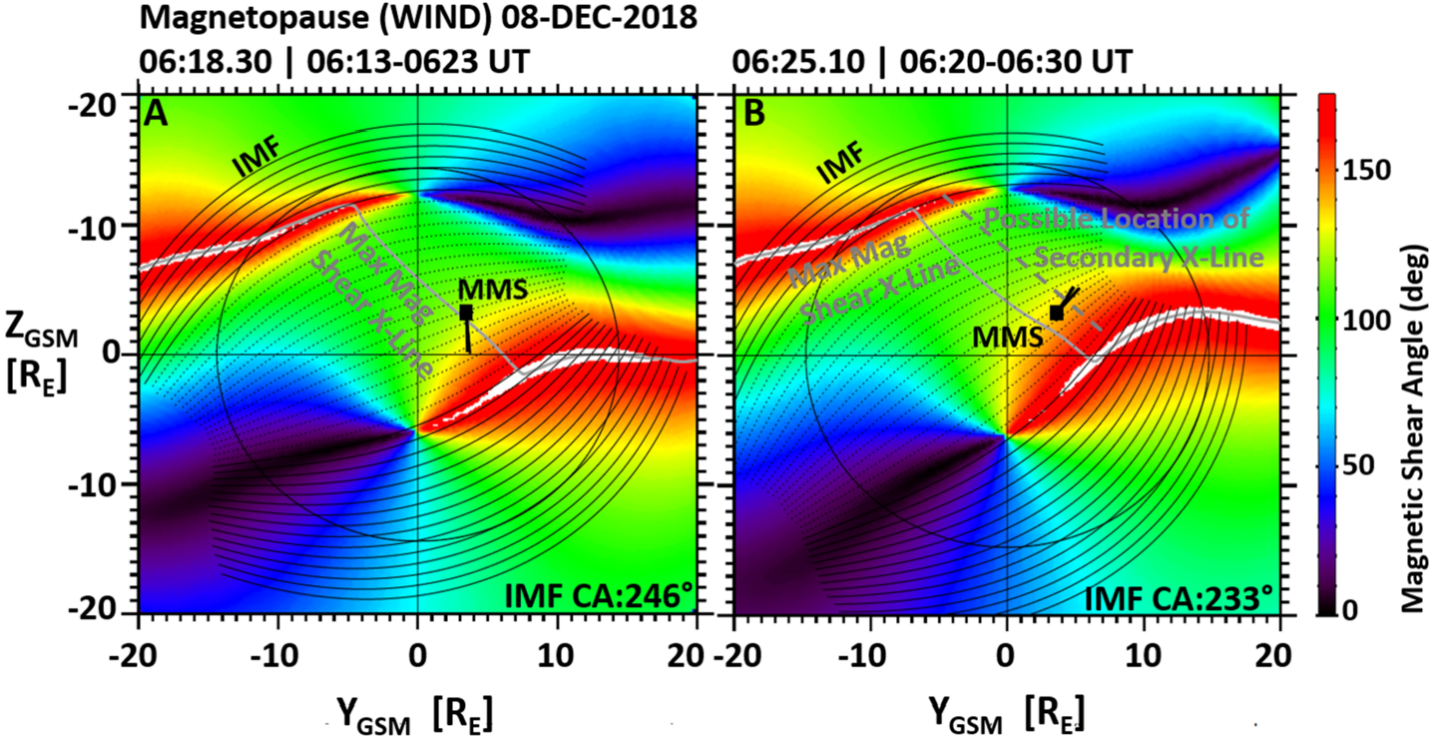
The magnetopause as viewed from the Sun towards the Earth, color-coded for the magnetic shear between the geomagnetic field (T96) and the draped IMF (curved black lines) (Kobel and Flückiger, 1994). Overlayed is the location of the MMS satellites at the magnetopause, observing an ion beam switch in the boundary layers and defining the location of the primary dayside reconnection line. Also shown is the predicted X-line location from the Maximum Magnetic Shear model (grey line) and the possible location of a secondary X-line (dashed grey line in the right panel)

Figure 6 shows the global impact of multiple X-lines. Plotted are the magnetopause magnetic shear maps for an MMS magnetopause crossing on December 8, 2018, at 06:18.30 UT (left panel) and 06:25.10 UT (right panel). Overlayed onto the magnetic shear maps are the predicted location of the magnetopause X-line (solid grey line) based on the maximum magnetic shear model (e.g., Trattner et al. 2007, 2021; Petrinec et al. 2014), the location of the MMS satellites at the magnetopause (black square symbol), the terminator plane separating the dayside magnetopause from the tail side magnetopause (black circle), and black curved lines representing the draped IMF lines from the Kobel and Flückiger (1994) model which is used together with the geomagnetic field lines from the T96 model (Tsyganenko 1995) to determine the magnetopause magnetic shear.

The MMS magnetopause crossing occurred about 2 hours before the TRICE-2 cusp crossing. Fortunately, the magnetopause crossing occurred under nearly the same solar wind and IMF conditions that were present during the cusp crossing (e.g., Fuselier et al. 2022; Trattner et al. 2024). In addition, as MMS crossed the magnetopause, it observed an ion beam “switch” in the magnetopause boundary layer, the signature of crossing an X-line (e.g., Cowley 1982; Gosling et al. 1982). The ion beam switch is depicted in Fig. 6 with the short black lines emanating from the MMS symbols that represent the Fast Plasma Instrument (FPI) plasma flow directions. This observed ion beam switch defines the location of the primary X-line at the magnetopause and agrees with the predicted X-line from the maximum magnetic shear model (e.g., Fuselier et al. 2022; Trattner et al. 2024).

The MMS ion observations in the magnetopause boundary layer also showed counter-streaming ion beams (not shown), which is the signature of secondary X-lines at the magnetopause. Since the solar wind and IMF conditions for the MMS magnetopause crossing and the TRICE-2 cusp crossing are the same, it confirms the presence of a temporary secondary X-line at the magnetopause for these input conditions. A possible location of this secondary line is depicted in Fig. 6.

Knowing the location of the dayside primary X-line can also be used to determine the plasma entry points of the precipitating ions in the cusp. As shown in Trattner et al. (2024), the rocket location in the cusp was used together with T96 magnetic field lines to trace back the precipitating ions to the magnetopause and connect to the known location of the magnetopause X-line, highlighting the plasma entry points for the ions observed in the cusp and documenting how these entry points moved as the rockets progressed through the cusp.

The slowest ions in a cusp distribution, defined in the literature as the 1/e cutoff velocity (e.g., Fuselier et al. 2000; Trattner et al. 2007, 2015) are coming from closest to the dayside X-line. The cutoff velocity (Ve), determined from cusp ion energy dispersions, together with the distance along the cusp magnetic field line to the known location of the dayside X-line (d), can be used to determine the “time-since-reconnection” ($\Delta $t = d / Ve) and subsequently the “reconnection time” for a cusp magnetic field line. This “reconnection time” is a direct measurement of continuous or pulsed reconnection as demonstrated in Trattner et al. (2015). A smooth rise of this time throughout the cusp shows continuous reconnection present at the magnetopause while a stepped rise of the time shows that pulsed reconnection is active at the magnetopause. Combining TRACERS ion observations with the X-line prediction model and the T96 model will provide this timing information and determine if reconnection is continuous or pulsed.

## Low Energy Ions in the Cusp

**Fig. 7:**
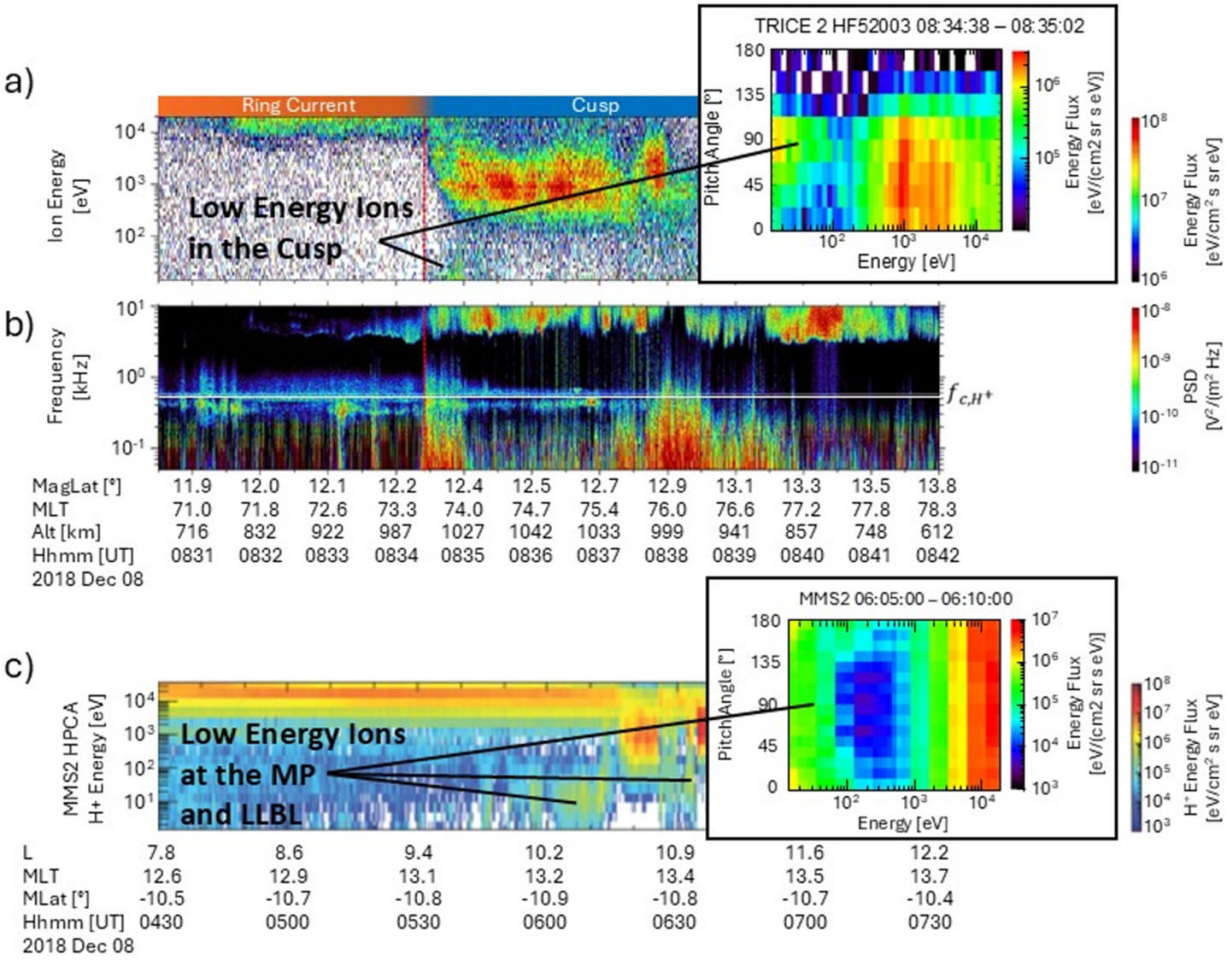
Observations obtained by the TRICE-2 High-Flyer (HF52003) sounding rocket and the MMS2 spacecraft. a) scaled energy flux for the ions measured by the TRICE-2 ESA, inset shows the pitch angle vs. energy distribution of the ions near the OCB; b) electric wave power spectral density (PSD) measured by TRICE-2 electric field instrument; c) energy flux of the H^+^ ions measured by MMS2/HPCA, inlet shows the pitch angle vs energy distribution of the ions near the magnetopause. The red line in Figure (a) depicts the OCB separating closed from open magnetic field lines. The white line in (b) denotes the proton cyclotron frequency (∼ 500 Hz). Adapted from Sawyer et al. (2021)

The synergy between multipoint observations throughout the magnetosphere was also used in a study of low energy ions observed in the cusp by the TRICE-2 rockets (Sawyer et al. 2021). Figure 7 (a) and (b) show the ion-energy time spectrogram and the electric field wave observations, respectively, obtained by the TRICE-2 High-Flyer rocket throughout the cusp traversal. While the rocket was on closed field lines (up to about 08:34.30 UT), ions at energies of 10’s of keV dominate the spectrum, consistent with ring current ions (e.g., Daglis et al. 1999; Fuselier et al. 2021; Sawyer et al. 2021). Upon crossing the OCB (marked by a thin red line in Fig. 7 (a)), ring current ions (10’s of keV), solar wind ions (around 1 keV), and a low-energy ion population (up to 200 eV) were observed (Chappell et al. 2008, Kitamura et al., 2010; Kistler and Mouikis 2016; Fuselier et al. 2016, 2017a).

The inset of Fig. 7 (a) shows the pitch angle distribution of the ion populations from 08:34.38 to 08:35.02 UT with the three ion populations occupying similar ranges of pitch angles between 0° and 180°. Pitch angles near 0° correspond to ions that are moving towards the Earth parallel to the magnetic field direction. Figure 7 (b) further shows that upon crossing the OCB, emissions consistent with broadband extremely low frequency waves (BBELF) (Bonnell et al. 1996; Bogdanova et al. 2004; Garbe et al., 1994; Shen et al. 2018; Bouhram et al. 2002; Kintner et al. 2000) were correlated with the observations of the low-energy ion populations. These waves were seen from near DC up to 2 kHz, encompassing the proton cyclotron frequency near 0.5 kHz.

Figure 7 (c) shows the MMS H^+^ ion-energy time spectrogram for when MMS was traversing the magnetopause nearly two hours prior to the TRICE-2 cusp traversal. The ion populations identified in the magnetopause low-latitude boundary layer (LLBL) and during the MMS magnetopause crossing from 06:15 to 06:50 UT are ions with energies of 10’s of keV, consistent with ring current ions, the magnetosheath distribution around 500 eV to 1 KeV, as well as a low energy ion population extending up to 200 eV. The inset of Fig. 7 (c) shows the pitch angle distribution of the ion populations from 06:05 to 06:10 UT on closed field lines. From the pitch angle distribution, the two ion populations that were observed near the magnetopause show the ring current near 10’s of keV uniformly occupying all pitch angles, while the low-energy ions at energies up to 200 eV have peaks at pitch angles of 0° and 180°, i.e. these ions are counter-streaming.

Previous studies of BBELF waves within the cusp have been attributed to multiple wave modes including Doppler-shifted dispersive Alfvén waves (e.g., Stasiewicz et al. 2000; Grison et al. 2005) and narrowband electromagnetic ion cyclotron (EMIC) waves (e.g., Erlandson et al. 1994, 2001, Grison et al. 2014), among other modes (See Shen et al. 2018). Shen et al. (2018) suggested multiple scenarios relating to localized heating processes driven by various wave modes that could produce an ion distribution similar to what was observed with TRICE-2. The localized heating processes that were explored were related to a narrow heating layer found near the F2 region of the ionosphere (Buchert et al. 2004), as well as a process similar to the “pressure cooker” phenomenon described by Gorney et al. (1985). Additionally, a process by which ion outflows may fall back towards the Earth was also proposed as a potential mechanism (Endo et al. 2000; Loranc et al. 1991; Ogawa et al. 2009). These localized heating scenarios seem unlikely in the context of the study by Sawyer et al. (2021) due to TRICE-2’s 1000 km altitude and the fact that no significant accompanying outflows were observed.

Sawyer et al. (2021) has suggested an alternate scenario based on the comparison of the TRICE-2 measurements in the cusp with the MMS observations near the magnetopause. Figure 7 (c) shows low-energy ions both near the magnetopause on closed field lines and within the LLBL on open field lines, while Fig. 7 (a) exhibited low-energy ions only upon crossing onto open field lines. Thus, it is probable that the counter-streaming low-energy ions observed on closed field lines had a mirror point at altitudes higher than the TRICE-2 rocket altitude. When TRICE-2 traversed the OCB, low-energy ions with an energy characteristic like those observed near the magnetopause and within the LLBL were observed. This led to the conclusion that the magnetic mirror points B_mp_ for these low-energy ions had lowered on the open magnetic field lines. Given the correlation between the BBELF wave spectrum missions and the presence of these low-energy ions in the low altitude cusp, pitch angle scattering via wave-particle interactions may be facilitating this lowering of the mirror point.

**Fig. 8:**
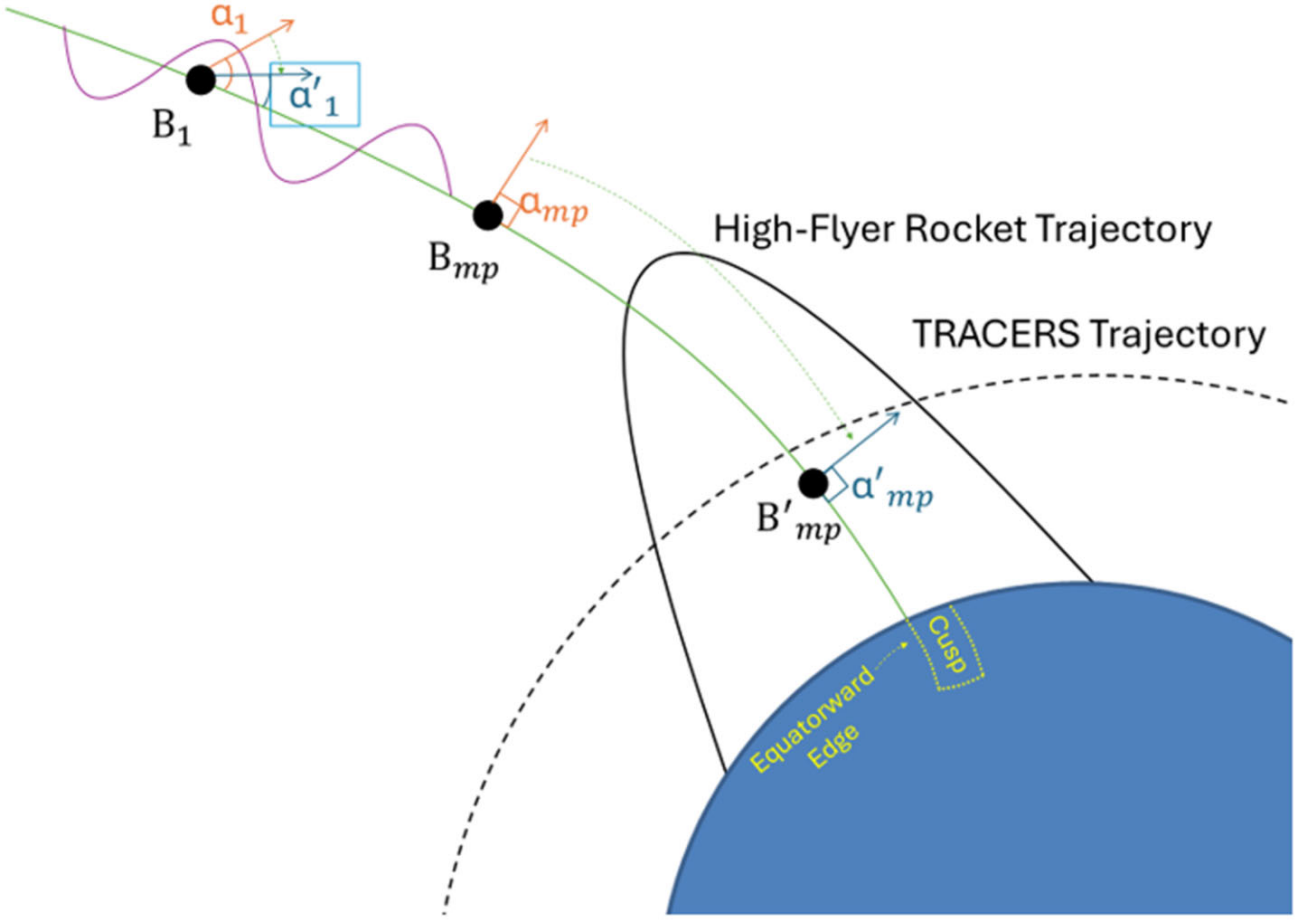
Schematic showing the effect of mirror point lowering due to pitch angle scattering of the low-energy ions. Wave-particle interaction changes the pitch angle for the precipitating ion from $\alpha _{1}$ to $\alpha _{1}$’. The magnetic mirror point lowers from the unprimed location at B_mp_ to the primed location at B’_mp_. Adapted from Sawyer et al. (2021)

Figure 8 shows a schematic whereby the ion pitch angle has been modified from B_mp_ to the B’_mp_ orientation, thereby causing the mirror point to shift to a lower altitude. In order to lower the mirror point, a change in the pitch angle of only a few degrees would provide a large shift of the mirror point to lower altitudes (e.g., Nykyri et al. 2019).

TRICE-2 has provided the groundwork upon which future missions and modeling efforts may be built upon. Further modeling efforts are required to explore the low-energy ion transport mechanism proposed in Sawyer et al. (2021); in particular an exploration of possible wave-particle interactions is warranted. TRACERS will be able to characterize these low-energy ion populations and answer questions related to the occurrence of similar low-energy ions within the cusp and whether there is a dependence on local time or solar wind conditions. With the aid of MMS in conjunction with TRACERS, it will also be possible to further explore the link between those low-energy ions near the magnetopause and those found within the cusp.

## TRACERS Auxiliary Science Inspired by TRICE-2 and Related Rocket Experiments

Jorgensen (1980), in a review of dayside auroral region electric fields, lists fifteen rocket flights probing dayside aurora prior to 1980 (cf., Table 1 of that article). In similar format, our Table 1 lists 25 rocket missions probing the dayside aurora since 1980, making more than 40 total flights (several missions involved two rockets). Not all of these flights penetrated the cusp, but many of them did. The early flights in Jorgensen’s (1980) table focused on establishing the existence of the cusp through detection of directly entered sheath plasma, along with the first evidence of accelerated electrons in the cusp, and associated convection measurements, in many cases using the barium jet technique. Later missions listed in Table 1 concentrated on convection patterns (#1, #8), ion outflow (#7, #9, #11, #13, #18, #23, #25), neutral upwelling (#17, #22, #24), waves and irregularities (#5, #14, #15, #18, #21), particle acceleration (#9, #20), and reconnection (#12, #19), although in many cases these experiments contributed to science topics different from their primary objective.

**Table 1:** Sounding rocket flights into the dayside aurora since 1980, complementing a similar table of pre-1980 flights previously published [Jorgensen 1980]. Location key: ARR=Andoya Research Range, CP=Cape Perry, NA=Ny Alesund, SS=Sondrestrom

#	Project	Location	Date	Time (UT)	Apogee (kms)	Example references
1	CENTAUR	CP	01/12/81	0138	612	Marklund et al. 1986
			13/12/81	2254	662	
2	CENTAUR	CP	07/12/81	0000	451	Christensen et al. 1986
3	CENTAUR	CP	06/12/81	2314	615	
4	CUSP	CP	21/01/82	0131	633	Boehm et al. 1984
5	COPE 1	SS	23/01/85	0923	500	Kelley and Earle 1988; Earle et al. 1989; LaBelle et al. 1989; Earle and Kelley 1993
6	GRNLD85	SS	23/01/85	0926:32	770	Boehm et al. 1990; Ergun et al. 1991
7	SCIFER	ARR	25/01/95	0624:48	1452	Kintner et al. 1996; Arnoldy et al. 1996; Pollock et al. 1996; Bonnell et al. 1997
8	36.153	NA	02/12/97	0842	430	Maynard et al. 2000
	36.152	NA	03/12/97	0906	447	
9	SS-520-2	NA	04/12/00	0916	1102	Tanaka et al. 2005
10	CUSP	NA	14/12/02	1116	768	Pfaff et al. 2004; Burchill et al. 2010
11	SERISIO	NA	22/01/04	0857	782	Frederick-Frost et al. 2007
12	TRICE	ARR	10/12/07	0900	1146	LaBelle et al. 2010
				0902	622	
13	SCIFER-2	ARR	18/01/08	0730	1468	Lund et al. 2012
14	ICI-2	NA	05/12/08	1035	329	Lorentzen et al. 2010; Moen et al. 2012; Oksavik et al. 2012; Spicher et al. 2015
15	ICI-3	NA	03/12/11	0721:31	354	Spicher et al. 2016; Jin et al. 2019
16	C-REX	ARR	24/11/14			
17	RENU-2	ARR	13/12/15	0734	447	Lessard et al. 2020
18	VISIONS-2	NA	07/12/18	1106	807	Takahashi et al. 2022
19	TRICE-2	ARR	08/12/18	0826	1042	Sawyer et al. 2021; Moser et al. 2021a; Fuselier et al. 2000; Petrinec et al. 2023
				0828	756	
20	CAPER-2	ARR	04/01/19	0927	774	Moser et al. 2021b
21	ICI-5	NA	26/11/19		252	
22	CHI	NA	10/12/19	0930	>350	Watanabe et al. 2020
23	SS-520-3	NA	04/11/21	1109	756	Ishisaka et al. 2023; Zushi et al. 2023
24	C-REX-2	ARR	01/12/21			Conde et al. 2023; Kwon et al. 2023
25	ENDURANCE	NA	11/05/22	0131	768	Collinson et al. 2024

Similarly, although reconnection, as probed by the TRICE sounding rocket missions (#12, #19), is the main focus of the upcoming TRACERS satellite mission, TRACERS will undoubtedly contribute to many of the other science investigations initiated by these ∼40 preceding sounding rocket flights. Three examples are: dayside large-scale whistler mode saucers possibly characterizing the cusp; the cusp as a laboratory for nonlinear wave processes; and use of wave diagnostics to measure ion composition. These types of studies also reinforce the primary TRACERS mission. For example, Langmuir waves in the cusp trace out the electron density profile which in the sounding rocket experiments helps identify the cusp. The wave phenomena also mark and help identify transitions in ion and electron distribution functions, including those related to reconnection.

### Dayside Whistler Mode Saucers Associated with the Cusp

An outcome of the recent CAPER rocket mission (#20 in Table 1) is the observation of unusual large-scale whistler mode saucers associated with the cusp (Moser et al. 2021a). Some examples of these were previously observed with the DEMETER satellite (James et al. 2012). Saucers result from whistler mode emission on the resonance cone from a concentrated source. The angular variation with frequency of the resonant whistler mode waves leads to a spatial pattern whereby higher frequencies are encountered further from the source and lower frequencies closer to the source, producing a “saucer” shape in the frequency-time spectrogram recorded by a spacecraft moving rapidly by the source (James 1976). Saucers are commonly observed in nightside aurora, typically though not always originating below the spacecraft and relatively small in spatial dimension. The unusual feature of the cusp saucers is that they originate far above the spacecraft (at altitudes of thousands of kilometers) and hence are quite large scale spatially (100s of kilometers) (James et al. 2012; Moser et al. 2021a).

A principal mystery raised by these events is the required stationarity of the source, which must also be small in size. Individual saucer arms persist for tens of seconds: nearly 100 seconds in some examples in Fig. 2 of Moser et al. (2021a) and up to 40 seconds in some examples in Fig. 6 of James et al. (2012). James et al. (2012) discusses a number of mechanisms for saucer generation, none of which fully explain the stationarity conundrum.

Regarding the large-scale dayside saucers, an open question is whether their source is the cusp.

The CAPER rocket evidence, such as tilting of the plane of polarization of the whistler mode waves as the rocket traversed near the cusp, strongly suggests a cusp source. Another open question is how common the large-scale dayside saucers are. The CAPER rocket flight provides a single example, and the DEMETER satellite mission provided relatively little data above 65 degrees latitude. There was evidence of a faint saucer arm in the TRICE-2 rocket data (#19 in Table 1). TRACERS can provide answers to these questions, informing the degree to which the saucer signatures can be used for remotely sensing the cusp or certain cusp conditions. Furthermore, establishment of a cusp source would inform identification of a generation mechanism for these saucer signatures which originate at high altitudes.

### Non-linear Waves/Wave Processes in the Cusp

Several types of possible nonlinear wave phenomena that characterize the cusp have been revealed by sounding rocket investigations. At high frequencies waves are consistently observed in the frequency band between the electron plasma frequency (f_pe_) and the upper hybrid frequency (f_uh_). Langmuir waves excited near f_pe_ with wavevectors parallel to the ambient magnetic field sometimes grow to large amplitudes, up to 1 V/m, and in the cusp these are strongly modulated leading to frequency fine structure (e.g., Ergun et al. 1991; Bonnell et al. 1997; LaBelle et al. 2010). Waves near f_uh_ are also sometimes highly structured (e.g., Moser et al. 2021b). At low frequencies, whistler mode signals are ubiquitous, including occasionally multiple simultaneous evenly spaced narrowband emissions with systematic time variation, which may result from linear or nonlinear processes.

**Fig. 9:**
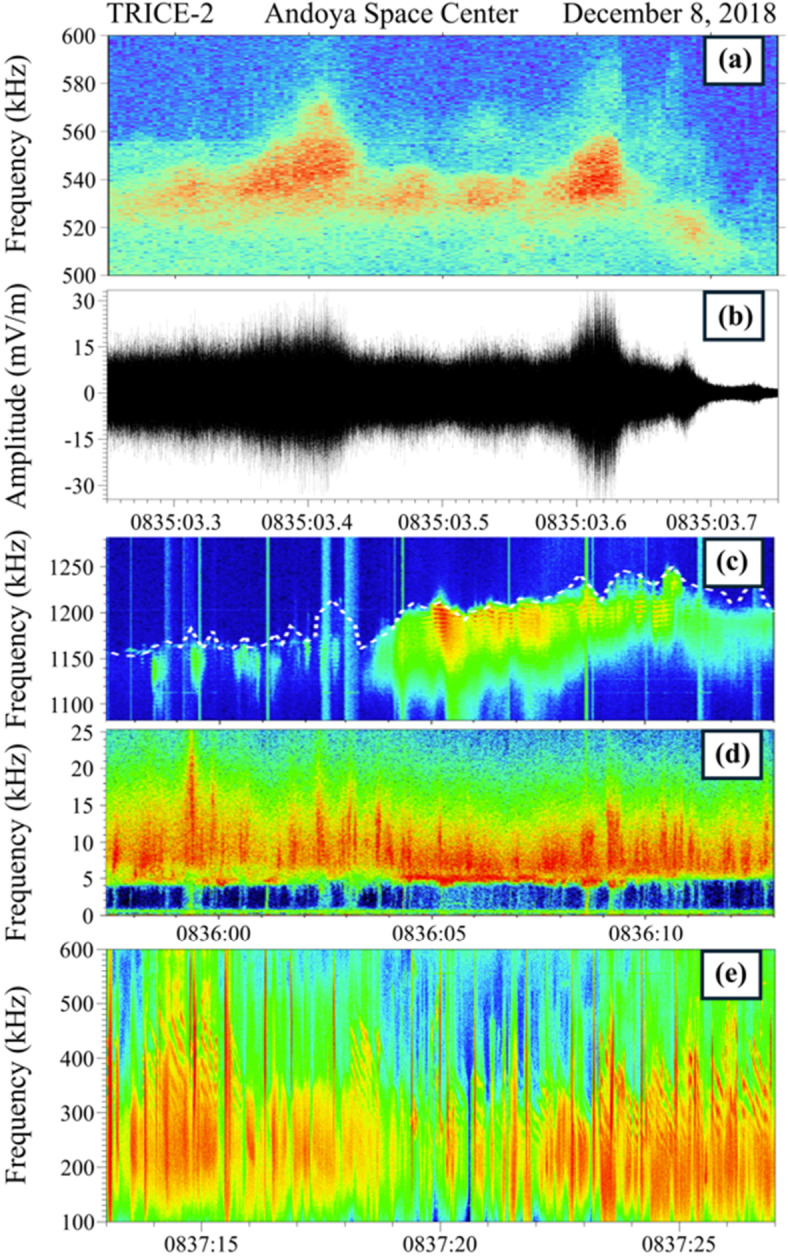
(a) Spectrogram and (b) corresponding waveform covering 0.5 s and 500-600 kHz showing structure near the plasma frequency measured with the TRICE-2 high flyer in the cusp ionosphere at 1026 km altitude; (c) HF spectrogram showing structured waves at and below the upper hybrid frequency, indicated by a white dashed line, and (d) VLF spectrogram showing structured waves near the lower hybrid frequency, measured with the TRICE-2 high flyer in the cusp ionosphere at 1042 km; (e) whistler mode “stripes” at 100-600 kHz measured with the TRICE-2 high flyer

Figure 9(a) shows a spectrogram representation of waves at and just below the plasma frequency, measured with the TRICE-2 sounding rocket during a 0.5-second interval at 1026 km altitude. Figure 9(b) shows the corresponding waveform, which is dominated by two approximately 50-ms duration wave bursts, one centered at 0835:03.4 UT and the other at 0835.03.62 UT. Langmuir waves in the cusp typically occur in bursts with duration 20-250 ms and with similar spectral form (e.g., Figs. 2 and 4 of LaBelle et al. 2010). The upper cutoff of the waves, ranging from 520-580 kHz in Fig. 9(a), tracks the plasma frequency. Each Langmuir wave burst corresponds to a localized plasma density enhancement which may result from impact ionization caused by the same filamentary electron beam that generates the Langmuir waves. Their integrated electric field amplitude, of the order of 15 mV/m, is within the range of Langmuir wave amplitudes previously recorded in the cusp (Ergun et al. 1991; Bonnell et al. 1997; LaBelle et al. 2010). Waves in the burst extend up to the maximum plasma frequency within the density enhancement and down to a frequency corresponding approximately to the ambient density outside the enhancements, similar to previous observations, except that in TRICE-2, discrete spectral peaks with few kHz separations arrayed below the plasma frequency are not strongly evident; the waves appear as more of a continuous spectrum. However, there is some frequency structure causing waveform modulations with periods of 50-150 microseconds, corresponding to roughly the 7-20 kHz spectral bandwidth.

Discrete spectral features below f_pe_ motivated a model whereby these are generated by wave-wave interactions between Langmuir waves and whistler/lower hybrid waves, shown to be kinematically possible and consistent with SCIFER sounding rocket data by Bonnell et al. (1997). Freja satellite data provided even stronger evidence for this process through direct observation of the putative VLF partner wave in some cases (Stasiewicz et al. 1996). However, in many cases the partner VLF wave is not observed, perhaps due to damping, propagation out of the region, or insufficient instrument sensitivity. An alternative hypothesis is independent linear excitation of Langmuir waves at different locations in the density enhancement corresponding to different plasma frequencies, followed by propagation of these into overlapping physical locations leading to wave beating and the observed wave modulation. This alternative mechanism would explain naturally why the waves extend only down to approximately the ambient plasma frequency and not further and is supported in some cases by the statistics of the wave amplitudes matching that predicted from modelling mixing of independent wave modes (Li et al. 2010). An open question is whether the independent Langmuir wave modes can propagate across the requisite distances without being damped away.

The TRICE-2 observations of a more continuous spectrum of waves within density enhancements between the ambient and enhanced plasma frequencies suggests a variation of the independent wave mode hypothesis wherein the Langmuir waves mode convert to whistler mode, which in the underdense domain (f_pe_ < f_ce_) of the TRICE and TRICE-2 High-Flyer observations extends up to the plasma frequency (f_ce_ is the electron cyclotron frequency). The generation of whistler modes through this mode conversion mechanism has been observed in AUREOL-3 satellite data (Beghin et al. 1989) and PHAZE-2 sounding rocket data (McAdams et al. 1999); in the latter case the mode converted waves manifested as “bands”, narrow-band emissions observed for tens of seconds emanating from “hot spot” Langmuir wave bursts. The whistler modes originating from Langmuir waves generated at different locations and plasma frequencies, propagating for a relatively long period of time with little damping, would eventually form a continuum of waves filling the density enhancement. Discrete features may be observed, as noted in the TRICE or SCIFER rocket observations, for cases in which relatively few sources contribute or in which not enough time has passed for multiple sources to contribute. Otherwise, a more continuous spectrum would be observed as in Fig. 9(a) and other cases as measured with the TRICE-2 and CAPER-2 rockets. The amplitude of the TRICE-2 waves (10-15 mV/m) is smaller than many of the examples previously observed and attributed to nonlinear processes (Ergun et al. 1991; Bonnell et al. 1997).

Open questions center around the viability of the wave mixing hypothesis and the relative importance and occurrence rates of either the linear or nonlinear mechanisms. TRACERS will encounter a large number of events, albeit in short snapshots similar to Freja and SCIFER observations rather than continuous waveforms as in TRICE and CAPER observations. Although the necessary high frequency magnetic component will not be observed, polarization measurements should provide evidence for reflecting or propagating waves. In addition, bicoherence or related multispectral analysis will be possible, and simultaneous VLF measurements should provide evidence for partner waves. TRACERS therefore will be well-equipped to determine the occurrence rates of different mechanisms giving rise to the Langmuir wave structure.

Figure 9(c), from Moser et al. (2021b), shows TRICE-2 observations of structured waves at and below the upper hybrid frequency. The structure manifests itself as multiple narrow-band peaks just below f_uh_ separated by about 5 kHz, which is approximately the lower hybrid frequency. Figure 9(d), also from Moser et al. (2021b), shows the corresponding simultaneous VLF whistler/lower hybrid waves. While broadband whistler mode hiss occurs continuously at frequencies at and above the lower hybrid frequency, the interval of multiple peaks below f_uh_ corresponds to the appearance of a distinctly different VLF wave, more intense and concentrated at the lower hybrid frequency. As shown by Moser et al. (2021b), the frequency of the lower hybrid waves matches the frequency separation of the spectral peaks below f_uh_. Furthermore, frequency variations in the former are mirrored in the latter, providing ‘smoking gun’ evidence of a relation between the two waves. Moser et al. (2021b) suggested that a nonlinear wave-wave interaction occurs which, based on the energy densities of the wave modes, is most likely a decay rather than a coalescence.

At least eight instances of multi-peaked structure at and below f_uh_ occur during the TRICE-2 rocket flight, ranging in duration from 0.5-3.0 s and spanning the time range 589-669 seconds after launch, corresponding to altitudes of 1030-1040 km (not all of this range is shown in Fig. 9(c)). The phenomenon was not observed in the TRICE-2 Low-Flyer, TRICE-1, or CAPER-2 rocket flights (of which only TRICE-1 reached above 1000 km). The occurrence rate of this phenomenon remains an open question, as is whether there is an altitude threshold and whether coalescence rather than decay is ever observed. The TRACERS waveform capture technique, similar to that employed in previous satellite missions such as Freja (e.g., Kintner et al. 1995), will be effective at answering these questions if the phenomenon occurs at the TRACERS altitude of 590 km.

Figure 9(e) shows TRICE-2 detection of structured whistler mode signals called “stripes” at 1042 km. These features have been previously observed in the nighttime aurora at altitudes of 600-740 km (Samara and LaBelle 2006; Colpitts et al. 2010). The slopes of the stripes observed by TRICE-2, approximately 167 kHz/s, are at the extreme low end of the range of slopes observed at lower altitudes in the previous flights. The time separation and maximum frequency, 400-500 kHz and 0.167 s respectively, are within the range of values previously observed. As in the previous observations, the “stripe” features are embedded within larger region of broadband auroral whistler mode hiss. In the nightside observations, the “stripes” are associated with regions of Alfvénically accelerated electron beams.

The generation mechanism for these features is uncertain. Samara and LaBelle (2006) noted that a concentrated source of whistler mode waves generated on the resonance cone at altitudes of 1300-3000 km and moving upward at 1000-5000 km/s would result in waves exhibiting the observed “stripe” dispersion at rocket altitudes. However, this mechanism would predict a several-degree offset in latitude between the magnetic field line of the observation and that of the source. Colpitts et al. (2010) investigated a mechanism whereby upward-going electron conics at altitudes far above the rocket, which have been associated with Alfvénic acceleration, generate Z-mode waves through cyclotron resonance which could mode-convert to whistler mode waves. Based on electron transport time, believed to be the governing timescale, this mechanism matches the slope of the stripe features and can explain their periodicity driven by the period of the causative Alfvén wave. However, a potentially significant problem with this mechanism is how to produce downward-going whistlers from the Z-mode waves which are generated perpendicular to the field line (Fig. 9 of Colpitts et al. 2010) and would refract upward in the upward-directed density gradient at the source altitudes of 2500-3500 km. A mode conversion mechanism would be needed to produce downward whistler modes from the upward Z-mode waves.

It would be highly desirable to detect the “stripe” features with an independent wave receiver differing from those used in the previous observations. The TRACERS snapshot strategy implies that it will detect the features as a banded emission without detecting the dispersion, but that is nonetheless a valuable confirmation.

### Cusp Diagnostics Using Plasma and Lower Hybrid Waves

Detection of both unstable and thermal plasma waves is a well-known method of measuring plasma density and temperature (e.g., Meyer-Vernet and Perche 1989), but it is also possible to extract ion composition information from wave data, in particular through detection of the lower hybrid frequency either as a discrete emission or a wave cutoff. Temerin and Kelley (1980) applied this method to waves measured in the cusp by sounding rockets launched to 500 km altitude from Sondrestrom, Greenland, in 1974-1975 (experiments #7 and #10 in the list compiled by Jorgensen (1980)). In this case, the determined lower hybrid frequency, combined with total density known from plasma frequency and Langmuir probe measurements, was used to determine the relative concentrations of $\mathrm{O}^{+}$ ions relative to heavier ions such as NO^+^ and $\mathrm{O}_{2}^{+}$. Evidence was found for an overabundance of heavier ions in the cusp between 150 and 400 km altitude. Temerin and Kelley (1980) proposed that this imbalance might result from conversion of O^+^ to NO^+^ in large electric fields observed in the same experiment.

**Fig. 10:**
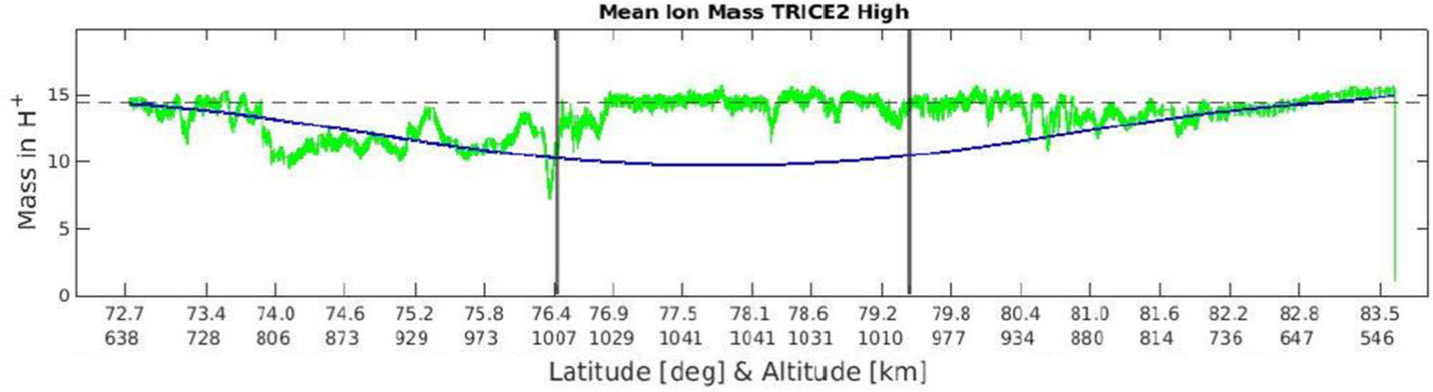
Mean ion mass M_eff_ in atomic mass units for a portion of the TRICE-2 high-flyer rocket flight, as a function of geographic latitude and altitude, calculated by using wave cutoffs to determine the plasma and lower hybrid frequencies. Adapted from Moser-Gauthier et al. (2024)

Since that time, both ion outflow and neutral upwelling have been identified as salient features of the cusp with significant implications for global space physics, motivating further development and applications of methods of obtaining information about cusp ion composition. The lower hybrid frequency may be defined in terms of a mean ion mass M_eff_ (e.g., Masson et al. 2009) through the relation

$$ \frac{1}{M_{\mathrm{eff}} ( m_{p} / m_{e} f_{lh}^{2} )} = \frac{1}{f_{pe}^{2}} + \frac{1}{f_{ce}^{2}} $$

where f_ce_ and f_pe_ are the electron cyclotron and plasma frequencies, respectively, and m_p_/m_e_ is the proton to electron mass ratio. The electron density, and hence f_pe_, is obtained from identifying the high frequency wave cutoff, similar to those shown in Figs. 9(a-e), with the electron plasma frequency. The magnetic field, and hence f_ce_, is known from magnetometer measurements. The lower hybrid frequency f_lh_ is inferred from the cutoff in the VLF hiss, similar to that shown in Fig. 9(d). Figure 10, adapted from Moser-Gauthier et al. (2024), shows the resulting mean ion mass in atomic mass units as a function of geographic latitude and altitude during a portion of the TRICE-2 high-flyer flight. Vertical lines delineate the approximate location of the cusp identified from particle and wave measurements. For reference, the mean ion mass predicted by the International Reference Ionosphere (IRI-2016) model (Bilitza et al. 2017) is shown with a blue line. Reminicent of the long-ago measurements of Temerin and Kelley (1980), the observed ion mass exceeds that predicted by IRI in the cusp region. The TRACERS electric field experiment will capture both the lower hybrid and plasma frequency cutoffs in waveform snapshots, enabling application of this technique to a huge number of cusp crossings under a wide range of conditions. A complete theory taking into account ion outflow, neutral upwelling, transport, and impact ionization will be needed to interpret the sounding rocket measurements or those obtained from TRACERS.

## Propagation of Dayside Magnetospheric Whistler Mode Emissions to Low Altitudes

Thanks to the unique two-point measurements of several components of the electromagnetic field fluctuations at a low altitude orbit, TRACERS will be also able to address several other long-standing problems of magnetospheric physics. Besides the relatively narrow region of magnetospheric cusps, the dayside magnetic field topology on the inner side of the magnetopause shows a direct influence of the solar wind on the magnetosphere. This topology of compressed field lines is then reflected by propagation of electromagnetic waves, which can travel over large distances and carry information about the plasma medium and magnetic field along their propagation path. TRACERS two-point measurements with the possibility of propagation and polarization analysis at each measurement point will be especially useful for investigating the ways that electromagnetic waves can propagate to low altitudes in the high latitude region.

Chorus and exohiss are important classes of magnetospheric whistler mode emissions in the audible frequency range occurring in the plasma trough region between the plasmapause and the magnetopause, with increased occurrence rates on the dayside (Li et al. 2009) over a large range of latitudes (Santolík et al. 2024). Chorus is observed with nonlinear discrete wave packets of changing frequency and with subpackets of rapid amplitude variations, but it can also coexist with exohiss without these nonlinear frequency-time structures. These waves directly influence the dynamics of the radiation belts. Energetic electrons can be accelerated locally by them (Horne et al. 2005) or precipitated into the atmosphere (Kasahara et al. 2018; Miyoshi et al. 2021). Chorus and exohiss waves are generated by wave-electron interactions close to the geomagnetic equator. This source location was confirmed by theoretical studies (e.g., Omura et al. 2008), by simulations (Katoh and Omura 2016), by in situ low-latitude observations (LeDocq et al. 1998), and by multipoint spacecraft measurements (Santolík et al. 2004).

Chorus emissions entering the plasmasphere at high latitude were also proposed to form an embryonic source of plasmaspheric hiss waves (Church and Thorne 1983), which are known for their strong effects on the radiation belts, causing losses of electrons especially from the slot region between the inner and outer radiation belts (Lyons et al. 1972). Ray tracing simulations (Bortnik et al. 2007; Hanzelka and Santolik 2019) of non-ducted propagation, however, need special initial conditions of oblique wave vectors in their equatorial source for the chorus waves to be able to propagate to high latitudes. This does not correspond to the most unstable wave vector directions, which are parallel to the local magnetic field lines (Kennel and Petschek 1966) and which would be attenuated in the magnetosphere before reaching the high latitude region (Bortnik et al. 2007).

**Fig. 11:**
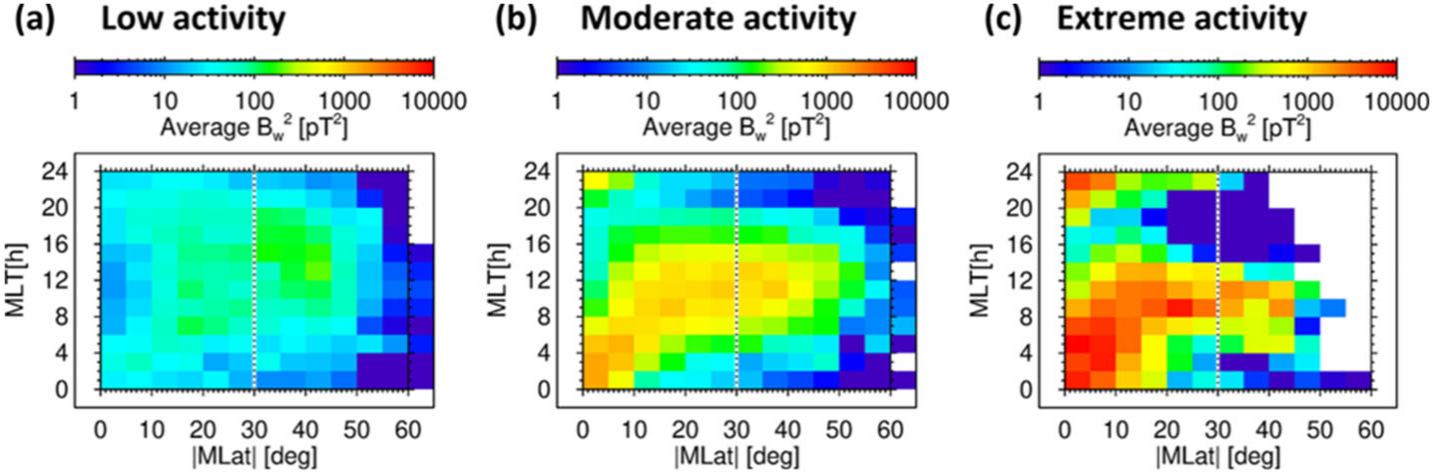
Long-term average squared amplitudes of whistler-mode chorus and exohiss as a function of the magnetic local time (MLT) and the absolute value of the geomagnetic latitude (MLat). Chorus and exohiss occur at higher latitudes on the dayside under different levels of geomagnetic activity, ranging from (a) low, with the AL* index above −100 nT, through (b) moderate, with the AL* index between −100 and −1000 nT, to (c) extreme, with the AL* index below −1000 nT. Adapted from Santolík et al. (2024)

Despite these simple theoretical predictions, spacecraft measurements show that whistler-mode waves systematically propagate to high latitudes (Fig. 11) on the dayside (Santolík et al. 2014, 2024), penetrating downwards into the upper ionospheric region (Santolik et al. 2006; Parrot et al. 2014) and to the ground (Storey 1953). A possible explanation may involve the presence of field-aligned density ducts (Bell et al. 2009; Hanzelka and Santolik 2019), which guide chorus whistler-mode waves to high latitudes and toward the Earth (Chen et al. 2021; Ke et al. 2021).

Joint observations of TRACERS (Miles et al. 2025a), the Observing Cusp Highaltitude Reconnection and Electrodynamics (OCHRE) sounding rocket (Powers et al. 2025) and ground-based stations, recording the electromagnetic waves in the audible frequency range, will thus be able to significantly contribute to investigations of the propagation of dayside magnetospheric whistler mode emissions to low altitudes.

## Magnetospheric Ducts and High Latitude Lightning

Occurrence and properties of field aligned density ducts themselves represent another long-standing problem which TRACERS can address. They are traditionally linked to propagation of lightning-generated whistlers (Storey 1953) and to their influence on magnetospheric particles through lightning-induced electron precipitation (Inan et al. 1989; Linzmayer et al. 2024). One of the possibilities for the embryonic source of plasmaspheric hiss proposed by Church and Thorne (1983) is also linked to the low-frequency component of whistlers that originate in lightning activity, consistent with multipoint spacecraft measurements (Santolík et al. 2021) of individual whistlers.

Many experimental studies show that whistler mode waves propagate over significant distances in magnetized plasma if guided by field-aligned density ducts (Chen et al. 2021; Williams and Streltsov 2021). Based on the transverse size of the inhomogeneity, the ducts can be considered narrow if they are less than 300 km wide, where the background magnetic field changes by less than 10%. In wide ducts, which were found to commonly range between 2000 and 7000 km wide based on a Van Allen Probes study (Williams and Streltsov 2022), both the effects of density variations and magnetic field changes play a role.

The existence of ducts has been routinely confirmed by in situ density measurements (Darrouzet et al. 2004; Moullard et al., 2004; Kurth et al. 2015). Another possibility for detecting ducts is through satellite or ground-based observation of whistler-mode waves propagating at very low wave normal angles. Observations from the Van Allen Probes were used to obtain the statistical wave normal distribution of lightning-generated whistlers (Xia et al. 2024). The authors found that lightning-generated whistler-mode waves trapped in ducts usually achieve larger amplitudes than waves outside the ducts, as they experience less Landau damping and more nonlinear growth during their propagation toward high latitudes. They also found that parallel propagation dominates in high L-shell regions, where the occurrence of lightning-generated whistlers is very low. At lower L-shells, the waves propagated mostly non-ducted. Direct evidence of ducts can be seen in lightning whistler echo trains: short ones observed by satellites in the magnetosphere (Smith and Angerami 1968) and long-lasting ones by ground-based receivers (Laaspere et al. 1965; Helliwell 1966).

**Fig. 12:**
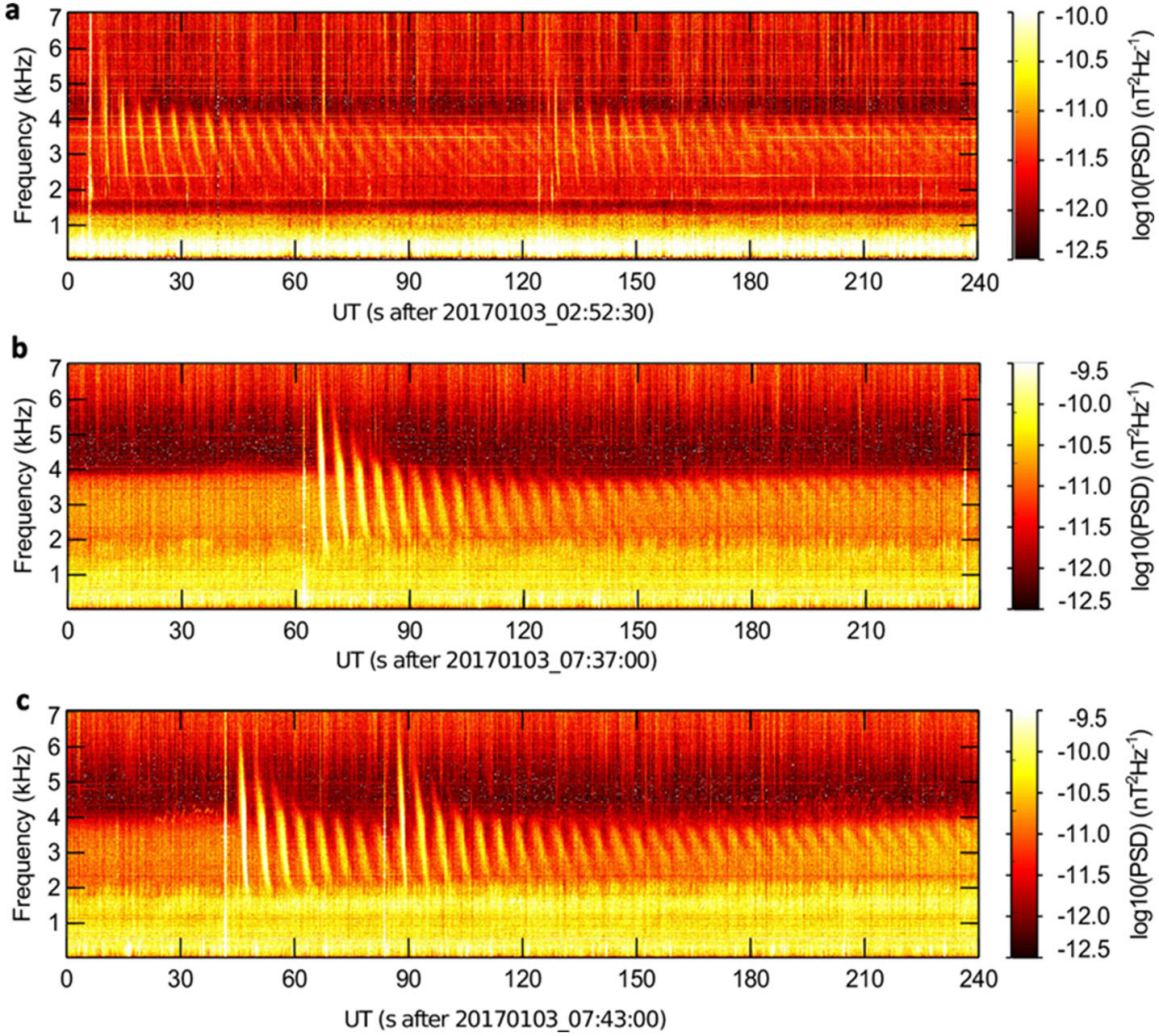
Three examples (a-c) of whistler echo trains recorded on January 3, 2017 by the high latitude receiving station Kannuslehto, Finland (67.7°N, 26.3°E). These frequency-time spectrograms show the power-spectral density of the magnetic field fluctuations at frequencies up to 7 kHz. Adapted from Kolmašová et al. (2024)

A recent study of long-lasting lightning whistler echo trains (Fig. 12) detected by a polar receiving station (Kolmašová et al. 2024) shows that the most intense whistler echo trains were produced by high-latitude lightning. This finding highlights the importance of high-latitude lightning, particularly the energetic ones that occur in winter, as a means to investigate duct properties. Holzworth et al. (2019) demonstrated a correlation between the fraction of strokes detected by the Worldwide Lightning Location Network (WWLLN) above 65°N and the global summer temperature anomaly over an 11-year period from 2010 to 2020. Moreover, the strokes producing the most energetic VLF sferics, known as superbolts with radiated energy above 1 MJ, tend to occur frequently in the North Atlantic, west of Europe.

For the above-mentioned reasons, TRACERS observations (Miles et al. 2025a), combined with OCHRE student sounding rocket measurements (Powers et al. 2025) and ground-based recordings, are ideal for investigating the characteristics of ducts using lightning whistlers produced by energetic high-latitude lightning discharges.

## Multi-Scale Observations of M-I Coupling: Combining TRACERS and AMPERE

While the primary science goal of TRACERS is to untangle spatiotemporal variability of dayside magnetopause reconnection through focusing on dynamics in the cusp, TRACERS will also observe electromagnetic fields and plasma dynamics, which are key to understanding high-latitude magnetosphere-ionosphere coupling. The two TRACERS satellites will measure the magnetic field perturbations associated with field-aligned currents (FACs), as well as the current-carrying particle populations, specifically precipitating electrons. Along each orbit, the TRACERS measurements are embedded in a global system of high-latitude FACs (i.e., the Birkeland currents) and currents associated with the cusp.

Combining TRACERS observations with the Active Magnetosphere and Planetary Electrodynamics Response Experiment (AMPERE) dataset will provide additional insight into magnetosphere-ionosphere (MI)-coupling processes. AMPERE measures the global, high-latitude FAC system in both hemispheres. This dataset is derived from magnetic field measurements from avionics magnetometers onboard the Iridium Communications satellite constellation in low-Earth orbit (Anderson et al. 2002, 2021; Waters et al. 2001, 2020). With continuous global coverage of magnetic field perturbations ($\delta\mathbf{B}$) and the resulting FACs, AMPERE observations allow for investigation of multi-scale dynamics around the northern cusp and provide contextual information for localized TRACERS measurements.

Additionally, one aspect of global context for TRACERS primary science that AMPERE can provide is an independent ability to track the location and motion of the OCB. The highest-latitude Birkeland currents (the region-1 sense) connect to the magnetopause, and so can be used to define the boundary between the open field lines of the polar cap and the closed field lines of the magnetosphere (e.g., Clausen et al., 2013). As shown in Fig. 2, understanding the large-scale configuration of the polar cap is key for understanding the cusp ion dispersion features.

**Fig. 13:**
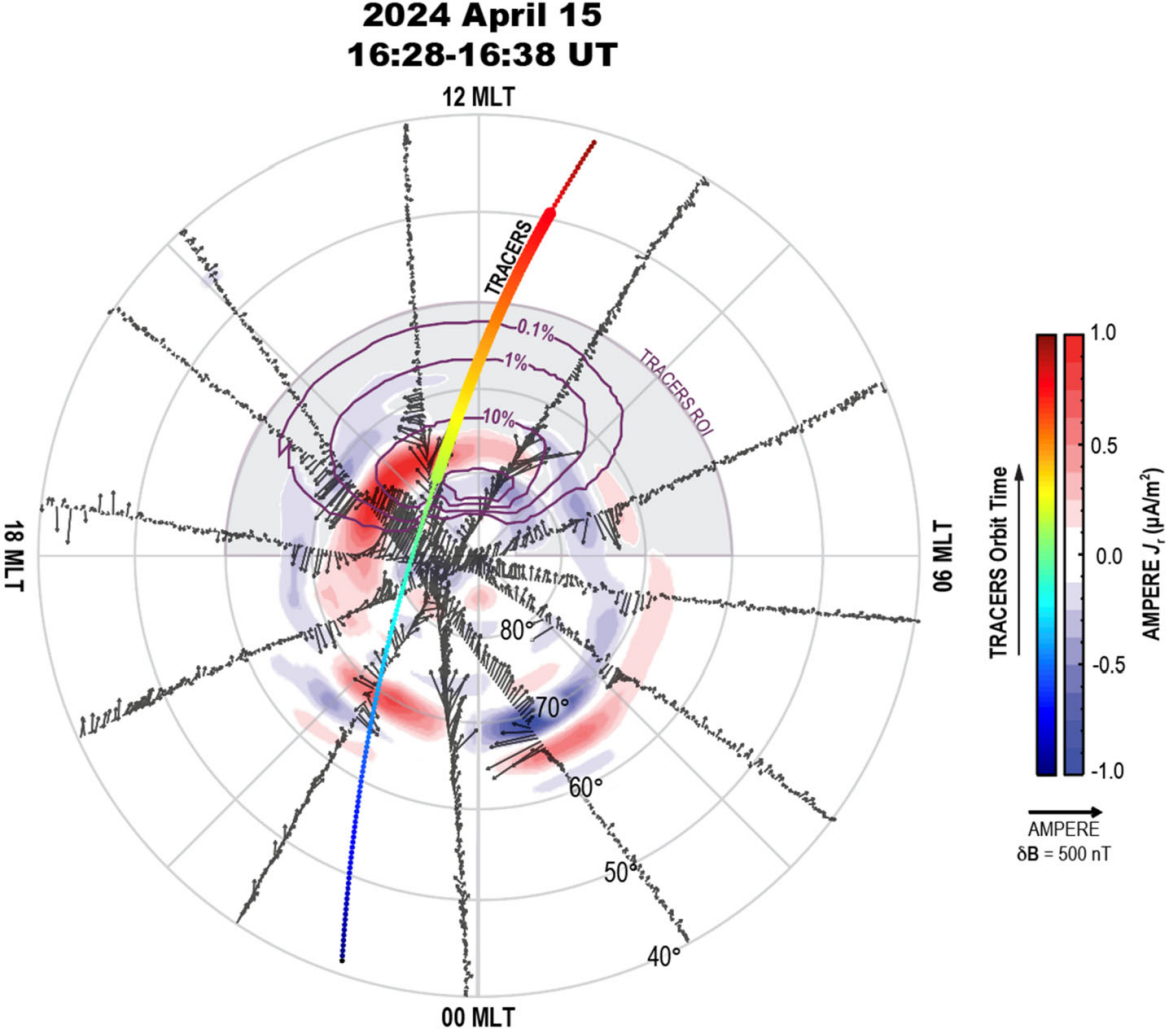
AMPERE horizontal field perturbations $\delta\mathbf{B}$ from the individual Iridium satellites in the northern hemisphere on 2024 April 15 from 16:28-16:38 UT. The resulting upward and downward FACs are shown by the red and blue contours, respectively. The probabilistic cusp location during this time interval is marked by the purple contours within the TRACERS region of interest (ROI, gray shaded area). Colored by time, the orbit of TRACERS starts from 40° latitude on the nightside, going through the polar cap to 40° on the dayside, with the thicker points marking the times coinciding with the AMPERE $J_{\mathrm{r}}$ distribution of 16:28-16:38 UT

Figure 13 shows an example of the TRACERS satellite trajectories through the northern dayside, high-latitude region with AMPERE horizontal perturbations $\delta\mathbf{B}$ and associated field aligned currents. This orbit corresponds to an interval of moderate upstream driving conditions near equinox, with an IMF clock angle of 215° and a dynamic pressure of about 2 nPa. During the time frame of this orbit, there are FACs observed by AMPERE, with both region-1 (R1) and region-2 (R2) sense currents developing into the well-known distribution associated with southward IMF: the R1 downward (upward) current spans the dawn (dusk) magnetic local times (MLTs) poleward of the R2 upward (downward) current (Iijima and Potemra 1976a,b; Le et al. 2010). Additionally, there is a region of downward current near 85°, poleward of the nominal R1 system, that stretches across the dayside from about 7-14 MLT. This portion of the current system arises from the contribution of the negative IMF B_Y_ component (e.g., Weimer 2001). The TRACERS orbit passes through these high-latitude dayside currents, which are co-located with the expected location and extent of the cusp for this time frame. How the local FACs that TRACERS will measure relate to the cusp ion signatures from spatiotemporal evolution of reconnection, and how that relates to global FAC dynamics, can be comprehensively investigated with TRACERS and AMPERE.

Along with determining the location, structure, and dynamics of the cusp within the larger-scale high-latitude current system, the combination of TRACERS and AMPERE measurements can be used to assess the contribution of localized plasma populations and currents to dynamics of the large-scale FAC system, especially during intervals of rapidly changing IMF B_Y_ and northward IMF. As noted in Cowley (2000), the current system that develops in response to IMF +B_Z_ is a result of reconnection poleward of the cusps and has a magnitude that is dependent on ionospheric conductivities (e.g., Fujii and Iijima 1987). Similarly, large, fast changes in the IMF B_Y_ component leads to reconfigurations of the FACs (e.g., Kunduri et al. 2020), with timescales and morphologies that are also dependent on the recent state of ionospheric conductance. AMPERE observations with repeated passes of TRACERS in similar local times will allow deeper investigation of the timescales of these reconfigurations in the FACs and polar cap convection patterns and how that relates to more localized variability in the cusp observed by TRACERS, as well as the plasma populations giving rise to changes in conductivities.

During intervals of northward IMF, the high-latitude dayside system appears to still be highly sensitive to slight changes in the upstream solar wind, producing complex auroral morphologies that indicate localized and rapidly evolving flows in the high-latitude ionosphere related to changes in open magnetic flux from the evolution of reconnection poleward of the cusp to dual-lobe reconnection (e.g., Frey et al. 2002, 2007; Milan et al. 2005, 2022; Carter et al. 2020; Hosokawa et al. 2020). However, cusp dynamics and high-latitude currents and convection during intervals of northward IMF are still not well understood (Fear 2021). It is difficult to understand the dynamics and drivers of auroral forms because of the lack of concurrent imaging with magnetic field and precipitating ion and electron observations at high enough spatiotemporal resolution. For example, using Imager for Magnetopause-to-Aurora Global Exploration (IMAGE) Far-Ultraviolet (FUV) observations in concert with Iridium engineering magnetometer data, Korth et al. (2004) identified a potential driving condition that gives rise to high-latitude dayside aurora (HiLDA) and is related to precipitation and the magnitude of FACs around the cusp. But, since the $\delta\mathbf{B}$ and resulting FAC observations were derived from Iridium engineering data prior to the standard AMPERE dataset (see also Anderson et al. 2000; Coxon et al. 2018; Vines et al. 2023), the required accumulation window of $\delta\mathbf{B}$ vectors was over one hour in time per distribution, necessitating that upstream conditions remain stable for several hours. While auroral imaging is currently still limited to small swathes from satellites like the Defense Meteorological Satellite Program (DMSP) and the Thermosphere Ionosphere Mesosphere Energetics and Dynamics (TIMED) missions (e.g., the DMSP passes used in Carter et al. (2020)) or to ground imagers, the current AMPERE data resolution combined with the high-resolution TRACERS magnetic field and plasma measurements will enable new, in-depth studies of the cusp dynamics and the relation to auroral signatures.

With electric and magnetic field measurements, TRACERS observations can also be used to derive other key parameters of ionospheric electrodynamics along the satellite orbits like Joule heating ($q_{\mathrm{J}}= \mathbf{J} \boldsymbol{\cdot} (\mathbf{E} + \mathrm{U} \times B)$, where $\mathbf{J}$ is the current density, $\mathbf{E}$ is the electric field, $\mathbf{U}$ is the plasma velocity, and $\mathbf{B}$ is the magnetic field) and Poynting flux ($\mathbf{S}= (\mathbf{E}\times \delta \mathbf{B})/\mu _{0}$), particularly the component parallel to the magnetic field ($S_{||}$) that indicates energy flow into the ionosphere. This can be used with AMPERE-derived electric potentials (e.g., AMPERE-MIX, from Merkin and Lyon 2010, using Robinson et al. (2020; 2021) conductivities) and/or Poynting flux (e.g., Billett et al. 2023; Chartier et al. 2022) to discern finer-scale structuring within the broader polar cap convection cells. Incorporation of other ground-based and satellite data (e.g., incoherent scatter radars and the SuperDARN network, SWARM, DMSP/SUSSI and TIMED/GUVI imaging, and ground-based magnetometers and all-sky imagers) can also be used in tandem with TRACERS and AMPERE for more comprehensive study of local to regional ionospheric convection patterns and electrodynamics. For example, Milan et al. (2022) investigated how FACs, ionospheric convection, and auroral forms in the polar cap evolve together over a period of long-duration northward IMF using AMPERE, SuperDARN, and DMSP, positing the role of reconnection in a “closed” magnetosphere in driving distinct flows and patterns of precipitation from the dayside to the nightside. Like studies using SWARM (e.g., Pakhotin et al. 2018), the high-resolution magnetic field observations from TRACERS in combination with the larger-scale AMPERE observations can also help discern the contributions of Alfvénic perturbations and particular wave modes in and near the cusp to the system electrodynamics and impacts on the underlying ionosphere (e.g., Lotko and Zhang 2018). TRACERS provides key measurements of local ion and electron populations that can further the understanding of the influence of instabilities in and near the cusp such as EMIC waves (e.g., Temerin and Lysak 1984; Santolík et al. 2002; Fuselier et al. 2004; Park et al. 2013; Pakhotin et al. 2022) and BBELF waves (e.g., Bonnell et al. 1996; Lund 2010; Sawyer et al. 2021), and how they relate to coupling of the cusp, outer magnetosphere to magnetopause, and ionosphere in the high-latitude dayside region.

Additionally, observations of local to global characteristics of Poynting flux and Joule heating are also key for understanding the impact of direct plasma entry from the cusp versus other sources of precipitation on the dynamics of ionospheric outflow, particularly the source location of O^+^ and dependencies on season, solar cycle, and upstream solar wind and IMF conditions (e.g., Pham et al. 2021; Zhang et al. 2013). Simulations of dayside outflow related to broadband electron and cusp precipitation were limited in their ability to fully resolve the cusp extent, depending on how the cusp was defined, and did not self-consistently account for underlying upper atmospheric dynamics like co-rotation (Pham et al. 2021). With TRACERS, AMPERE, and the multitude of ground-based and satellite remote sensing observations of the high-latitude ionosphere, substantial progress will be made in understanding the impacts of cusp dynamics on the coupled magnetosphere-ionosphere-thermosphere system.

## Summary and Discussion

Magnetic reconnection (e.g., Dungey 1961, 1963; Gonzalez and Mozer 1974; Paschmann et al. 1979; Sonnerup et al. 1974, 1981; Crooker 1979; Luhmann et al. 1984; Cowley and Owen 1989; Gosling et al. 1990) is the central process that drives the dynamics of solar-terrestrial interactions, allowing solar wind plasma to cross the magnetopause and enter the Earth’s magnetosphere. While decades of research, and especially recent in-situ observations by the MMS mission (e.g.; Burch et al. 2014, 2016; Cassak and Shay 2007, 2008; Cassak and Fuselier 2016; Fuselier et al. 2017a; Webster et al. 2018), have revealed many details of the magnetic reconnection process, some of the more global aspects have remained elusive.

Solar wind ions injected at the magnetopause stream along the newly opened geomagnetic field lines into two narrow polar regions known as the magnetospheric cusps. Within the cusps (e.g., Burch 1972; Newell and Meng 1992; Woch and Lundin 1992; Lavraud et al. 2005; Trattner et al. 2002; Merka et al. 2005), the global consequences of magnetic reconnection are elucidated by studying their respective signatures (e.g., Rosenbauer et al. 1975; Shelley et al. 1976; Cowley et al. 1991; Cowley and Lockwood 1992; Lockwood and Smith 1994; Oieroset et al. 1997; Fuselier et al. 1999; Trattner et al. 2005b; Escoubet et al. 2008), which are left in the precipitating ions arriving from the magnetopause reconnection site. The TRACERS mission, with the overarching goal to connect the magnetospheric cusps to the magnetopause and resolve the global aspects of magnetic reconnection, will observe these cusp structures and address the following science objectives:

1. Determine whether magnetopause reconnection is primarily spatially or temporally variable for a range of solar wind conditions.
2. For temporally varying reconnection, determine how the reconnection rate evolves.
3. Determine to what extent dynamic structures in the cusp are associated with temporal versus spatial reconnection.

There is no doubt that magnetic reconnection is both spatially and temporally variable. However, the characterization of the temporal (e.g., Cowley and Owen 1989; Lockwood and Smith 1989, 1990; Escoubet et al. 1992; Lockwood et al. 1998) or spatial nature (e.g., Weiss et al. 1995; Onsager et al. 1995; Trattner et al. 2002, 2005a) of magnetic reconnection was previously complicated by the fact that chance conjunctions of satellites in the cusp are not usually in the same local time sector and are also separated in time by tens of minutes or even hours. Despite these significant limitations, several multi-satellite event studies show remarkable similarities in their respective cusp ion-energy profiles (e.g., Onsager et al. 1995; Trattner et al. 2005b) which have been interpreted as spatial cusp structures. The TRACERS satellites will cross the cusp in 1-2 minutes in the same local time sector and provide true snapshots of the cusp ion-energy profile for comparison, in order to determine if magnetic reconnection at the Earth’s magnetopause is dominated by temporal or spatial effects. The results will be combined with the associated solar wind input conditions to determine the driving parameters for either reconnection scenario.

For temporal magnetic reconnection cases, the variability of the reconnection rate throughout the cusp crossing will be analyzed using methodologies developed by Lockwood and Smith (1992, 1994) and Lockwood et al. (2005). The first method uses measurements by the electric field experiment on TRACERS to determine the total convection velocity of the plasma across the open-closed field line boundary. The total convection velocity is the vector sum of the reconnection inflow velocity (the reconnection rate), the satellite velocity in the cusp (from ephemeris data) and the motion of the open-closed field line boundary (e.g., Lockwood 1995). The motion of the open-closed field line boundary is determined by comparing the location of the boundary encountered by each TRACERS satellite. What remains are two instantaneous measurements of the inflow velocity across the open-closed field line boundary, which is the instantaneous reconnection rate (e.g., Lockwood et al. 2005).

The second method can be applied to cusp observations of a single spacecraft (Lockwood and Smith 1992, 1994) and uses the low-velocity cutoff of the cusp ions (e.g., Onsager et al. 1990, 1991; Fuselier et al. 2000; Trattner et al. 2007, 2021) to determine the time history of the reconnection rate as the spacecraft traverses the cusp. To determine how the magnetic reconnection rate evolves, the relative reconnection rate E_Y_ is given by Lockwood and Smith (1992):

$$ E_{Y} =( B_{i} V_{sc} \cos \alpha )(1+ \left ( \frac{d}{2} \right ) \left ( \frac{m}{2} \right )^{1/2} E_{ic}^{-\frac{3}{2}} \frac{d E_{ic}}{dt} )^{-1} $$

where B_i_ is the ionospheric magnetic field (from TRACERS magnetic field measurements and the T96 model), V_sc_ is the spacecraft velocity (from ephemeris data), $\alpha $ is the angle between the reconnection flow and the spacecraft velocity (determined from TRACERS electric field measurements), $m$ is the cusp ion mass (assumed to be protons; $m = 1$ amu), $d$ is the distance to the reconnection X-line at the magnetopause (determined using the maximum magnetic shear and T96 models), E_ic_ is the ion low-velocity cutoff energy and dE_ic_/dt the change in this cutoff energy across the cusp (determined from cusp ion spectrograms).

The reconnection rate as given by Lockwood and Smith (1992) covers many different observation scenarios; including whether the satellite velocity is either slower or faster than the convection velocity of the magnetic field lines in the cusp. Different satellite velocities directly influence the value of dE_ic_/dt in the cusp. For stable solar wind and reconnection conditions at the magnetopause, a slow-moving satellite will observe small changes in the time-since-reconnection of the convecting magnetic field lines and therefore observe a flat profile for dE_ic_/dt (i.e., |dE_ic_/dt| ∼ 0). In contrast, a satellite that crosses quickly onto older open magnetic field lines with larger changes in the time-since-reconnection will observe a steep profile for dE_ic_/dt (i.e., large value of |dE_ic_/dt|). TRACERS will always be moving fast compared to the convection velocity of the magnetic field lines and in the opposite direction for southward IMF conditions. Therefore, in the case of TRACERS cusp traversals during southward IMF, the value of dE_ic_/dt is expected to be large and negative. A special case of temporal reconnection involves the conditions that cause continuous steady-state reconnection at the magnetopause and no cusp steps in the ion dispersion profile, which will be documented by the TRACERS mission.

Observations from the TRACERS mission will provide several auxiliary science options that will contribute to the science objectives, in addition to collaborations with ongoing observations campaigns in a synergistic way, by combining multiple platforms to provide a global view of the magnetic reconnection process and impacts on the coupled magnetosphere-ionosphere system. Such an approach was recently used by Trattner et al. (2024), who combined rocket (TRICE 2), satellite (MMS) and ground-based observations (SuperDARN), with models (maximum magnetic shear prediction model and the T96 (Tsyganenko 1995) geomagnetic field line model) to study plasma entry into the magnetosphere, especially spatial versus temporal magnetic reconnection. This approach allowed for multiple perspectives of an interval of large-scale magnetic reconnection. The combined observations provide the plasma entry points at the magnetopause, their spatial extent along the X-line, global context of the surrounding ionospheric convection, and details of precipitating ions deep within the cusp.

Due to its high cadence of cusp observations, the TRACERS mission will establish the relative occurrence rate of multiple X-lines at the magnetopause by documenting overlapping ion-energy dispersions in the cusp and determining the associated solar wind and IMF conditions (e.g., Lee and Fu 1985; Fuselier and Lewis 2011; Fuselier et al. 2018, 2022; Vines et al. 2017; Trattner et al. 2012, 2024; da Silva et al. 2024). Another methodology used by the TRACERS mission will determine the “reconnection time” of the cusp magnetic field lines at the magnetopause using the low-velocity cutoff velocity from the ion observation and the known distance to the primary dayside X-line (e.g., Trattner et al. 2015). The profile of this reconnection time is a direct measurement of the pulsed or continuous nature of magnetic reconnection.

Based on the TRICE-2 results, TRACERS will also explore the low-energy ion transport mechanism based on wave-particle interaction proposed by Sawyer et al. (2021) and characterize the low-energy ion populations to establish whether there is a dependence on local time or solar wind conditions.

The TRICE-2 sounding rocket mission and others listed in Table 1 inspire a host of studies that will be significantly advanced by TRACERS. For example, TRACERS will detect large-scale VLF saucer features on the dayside and answer the question of whether these are associated with the cusp and how frequently if so. TRACERS will also measure many structured Langmuir and upper hybrid waves during many different cusp crossings under different conditions and will answer how often linear versus nonlinear processes are responsible for these, and if nonlinear how often decay versus coalescence occurs. For a third example, by detecting the electron plasma and lower hybrid frequencies from wave cutoffs, combined with magnetometer measurements providing the electron cyclotron frequency, TRACERS will obtain ion composition information at intervals across thousands of cusp crossings to inform modeling of ion outflow and neutral upwelling processes. These examples related to plasma waves give a hint at the breadth and depth of auxiliary space plasma physics studies enabled by the TRACERS mission. Furthermore, these studies relate to the main TRACERS mission since the various wave phenomena provide measurements of electron density as well as transitions in particle distribution functions that assist in identification of the cusp and its features.

Both TRACERS spacecraft will carry a suite of instruments (Halekas et al. 2025; Fuselier et al. 2025; Bonnell et al. 2025; Hospodarsky et al. 2025; Strangeway et al. 2025; Miles et al. 2025b; all in this collection), which will perform multicomponent measurements of electromagnetic waves in the audible frequency range. These unique two-point measurements will bring new results on wave propagation, relevant to several open questions of magnetospheric physics. For example, occurrence and properties of density ducts in the magnetosphere can be investigated by analysis of lightning generated whistlers recorded by TRACERS. Their measurements also contribute to the analysis of effects of whistlers on precipitation of energetic electrons from the radiation belts. Waves generated by plasma instabilities in the equatorial region can, in turn, accelerate electrons to relativistic energies. If these waves propagate down to the low-Earth orbit at high latitudes, TRACERS will detect them and contribute to their investigation by analyzing their polarization and propagation properties.

TRACERS also provides high-resolution measurements that are embedded in multi-scale systems of the high-latitude magnetosphere-ionosphere coupling region. For example, the two TRACERS satellites will measure the magnetic field perturbations associated with FACs, electric fields, and waves that contribute to energy transfer (e.g., via Poynting flux and Joule heating) and plasma motion, as well as the precipitating populations that give rise to changes in conductivities and auroral forms. Together with AMPERE and the multitude of ground-based and satellite remote sensing observations of the high-latitude ionosphere, TRACERS will enable substantial progress in understanding the impacts of dayside magnetopause reconnection and cusp dynamics on the coupled magnetosphere-ionosphere-thermosphere system.

## Data Availability

The TRICE-2 data are available from the University of Iowa at the following website: https://space.physics.uiowa.edu/rockets/data/SCIENCE/TRICEII_Mission/. Data sets from the Magnetospheric Multiscale (MMS) mission are available through the MMS Science Data Center (https://lasp.colorado.edu/mms/sdc/). Interactive Data Language (IDL) routines for display of MMS and TRICE-2 data are available in the current Space Physics Environment Data Analysis Software (SPEDAS) software package, which can be found through the MMS Science Data Center and through the SPEDAS website (http://spedas.org). Solar wind observations are provided by the Wind “Solar Wind Experiment” (Wind/SWE) (Ogilvie et al. 1995). The interplanetary magnetic field (IMF) measurements are provided by the Wind “Magnetic Field Instrument” (Wind/MFI) (Lepping et al. 1995). The solar wind data are available at the Coordinated Data Analysis Web (CDAWeb; http://cdaweb.gsfc.nasa.gov/istp_public/). The authors acknowledge the use of SuperDARN data. SuperDARN is a collection of radars funded by the national scientific funding agencies of Australia, Canada, China, France, Italy, Japan, Norway, South Africa, the United Kingdom, and the United States of America. SuperDARN RawACF data were obtained via setting up a user account for the British Antarctic Survey Data mirror (https://www.bas.ac.uk/project/superdarn). The Radar Software Toolkit (RST) to process the SuperDARN data can be downloaded from Zenodo (https://doi.org/10.5281/zenodo.1403226). Due to the nature of the account setup, data may not be accessible immediately. This research used the SPECTRE High Performance Computing Facility at the University of Leicester.
